# A versatile nanoplatform with excellent biofilm permeability and spatiotemporal ROS regulation for peri-implantitis treatment

**DOI:** 10.7150/thno.108830

**Published:** 2025-02-24

**Authors:** Zeyu Han, Ying Li, Xin Zhan, Ming Sun, Yan Liang, Mujie Yuan, Yong Sun, Jie Cao, Baodong Zhao, Fan Li

**Affiliations:** 1 Department of Oral Implantology, The Affiliated Hospital of Qingdao University, Qingdao University, Qingdao 266000, P. R. China.; 2 School of Stomatology, Qingdao University, Qingdao 266000, P. R. China.; 3 Department of Pharmaceutics, School of Pharmacy, Qingdao University, Qingdao 266021, P. R. China.

**Keywords:** peri-implantitis, biofilm penetration, antibacterial photodynamic therapy, reactive oxygen species, macrophage polarization

## Abstract

**Rationale:** Dental implant restoration is essential for rehabilitating dentition defects. However, peri-implantitis (PI) seriously threatens the long-term stability of implants. Treating PI requires the complete eradication of plaque biofilm and the meticulous modulation of inflammatory responses. Antibacterial photodynamic therapy (aPDT) presents a promising potential in the antibacterial realm. Nonetheless, traditional aPDT for PI faces challenges such as inadequate biofilm penetration and distribution of photosensitizers, as well as a lack of precise bacteria targeting. Moreover, the excessive ROS generated by aPDT will aggravate the oxidative stress of peri-implant tissues, and this issue cannot be neglected.

**Methods:** The CuTA-Por@ε-PL nanoplatforms (CPP NPs) were synthesized and characterized using dynamic light scattering, transmission electron microscopy, and dye probes in detail. The antibacterial and anti-inflammatory activities of CPP NPs were evaluated both *in vitro* and *in vivo*. Moreover, the *in vivo* therapeutic efficacy was successively analyzed through micro-CT, hematoxylin and eosin staining, Masson's staining, immunofluorescence staining, and colony formation units (CFU), among other techniques.

**Results:** Porphyrin (Por), CuTA nanozyme with SOD/CAT activities, and ε-Polylysine (ε-PL) were combined to fabricate CPP NPs via a straightforward approach. The notable positive charge of CPP NPs facilitated biofilm penetration, distribution and precise bacteria targeting. Then, irradiation with a 660 nm laser triggered a ROS burst for biofilm elimination. After aPDT, CPP NPs scavenged the residual ROS and modulated host immunity by regulating macrophage polarization. As a result, CPP-treated groups demonstrated the most outstanding antibacterial and anti-inflammatory performance in the rat PI model.

**Conclusions:** Given the pathogenesis of PI, this strategy rationally designed a multifunctional NP with antibacterial and anti-inflammatory functions via spatiotemporal ROS regulation. It provides a potentially novel approach for PI treatment, which may have a profound impact on improving the prognosis of patients with PI and advancing the field of implant dentistry.

## Introduction

Dental implants, known as "the third set of teeth for human beings", have become the main restoration method for dentition defects or losses, effectively restoring chewing function and aesthetics and improving the quality of life of tooth-missing patients 1-3. Their long-term stability depends on multiple factors, with peri-implantitis (PI) being the leading cause of stability loss 4, 5. PI has a high prevalence (19.53% at the patient level and 12.53% at the implant level) 6, and its cases are expected to increase as implant placements rise. PI is a multi-factor, inflammatory and infectious disease, with plaque biofilm as the initiating factor, leading to excessive inflammation and tissue destruction 7. Traditional treatment, mainly mechanical debridement with antibiotics, is limited by the peri-implant pocket's structure and antibiotic resistance 8-10, and frequent mechanical debridement can damage the implant surface, causing bacterial recolonization and biofilm reformation 11.

Recently, antibacterial photodynamic therapy (aPDT) has attracted attention for its advantages like minimal invasiveness and lack of bacterial resistance 12, 13. Some photosensitizers (PS), like methylene blue, porphyrin (Por), Chlorin e6 (Ce6), possess the capacity to instantaneously and abundantly generate reactive oxygen species (ROS) by photocatalysis under the excitation light irradiation 14, 15. They have been applied in the treatment of PI or periodontitis, and have attained certain clinical outcomes 16, 17. Additionally, recent evidence-based reviews imply that ROS adjunctive therapy may procure more favorable outcomes in reducing the number of Porphyromonas gingivalis (*P. gingivalis*) around implants, diminishing pocket depth and augmenting tissue attachment in patients afflicted with PI in comparison to conventional PI treatment modalities 18.

However, prior to the clinical application of aPDT considering the tissue characteristics of peri-implant diseases, several difficulties remain to be addressed. First, the protection provided by extracellular polymeric substances (EPS) in biofilms hinders the penetration and dispersion of PS, making it difficult for PS to permeate into the interior of biofilms, leading to insufficient antibacterial efficacy 19. Second, most PS currently lack specific targeting ability for bacterial biofilms, implying that PS not only act on target bacterial biofilms but also have non-specific phototoxic effects on surrounding normal tissues 20. This is a crucial concern for PI tissues already in a state of excessive oxidative stress. Third, ROS is a double-edged sword in aPDT. The elimination of plaque biofilm depends on significant ROS release, yet excessive ROS induces proinflammation and oxidative stress in surrounding normal tissues 21. This has been overlooked and severely impedes clinical translation.

Nanozymes with superoxide dismutase/catalase (SOD/CAT) activities possess the capability to scavenge ROS and subsequently produce water and oxygen 22, 23. The integration of SOD/CAT nanozymes and PS has emerged as an efficacious strategy for regulating the generation and elimination of excessive ROS. In the research conducted by Sun *et al.*, the PS Ce6 was combined with CeO_2_ nanozyme possessing ROS scavenging ability based on SOD/CAT enzyme activities. Through the spatiotemporal control of ROS generation and elimination, a favorable therapeutic effect on periodontitis was achieved 24. Copper tannic acid coordination nanosheet (CuTA NS), a new nanozyme with SOD/CAT activity, has shown effectiveness in treating diabetic wounds and periodontitis 25. Based on the research findings, CuTA has manifested remarkable effectiveness in treating diabetic wounds and periodontitis due to its SOD/CAT enzyme activities 26, 27. Furthermore, similar to CeO₂, a substantial quantity of hydroxyl groups are present on the surface of CuTA, which is conducive to the implementation of surface modification 28. Hence, we hypothesized that the strategy of combining CuTA with PS could realize a treatment mode of “generating ROS triggered by laser irradiation for antibacterial effect and scavenging residual ROS generated by aPDT without laser irradiation for anti-inflammatory effect”, thereby avoiding the side effects of aPDT.

However, the water solubility of modified metal-coordinated or metal oxide nanozymes is often unsatisfactory, which limits their penetration in biofilms 29, 30. ε-Polylysine (ε-PL), a cationic polymer generated via bacterial fermentation, can substantially augment the water solubility of negatively charged metal-coordinated nanozymes, thus enabling the attainment of an appropriate hydrated particle size 31, 32. Additionally, during the interaction with biofilms, the positive charges carried by ε-PL can attract the negative charges present on the bacterial surface. This electrostatic interaction is beneficial for enhancing the penetration and bactericidal efficacy of nanoparticles, such as nanozymes or PS-modified nanozymes, against biofilms and bacteria 33.

**Scheme 1:**
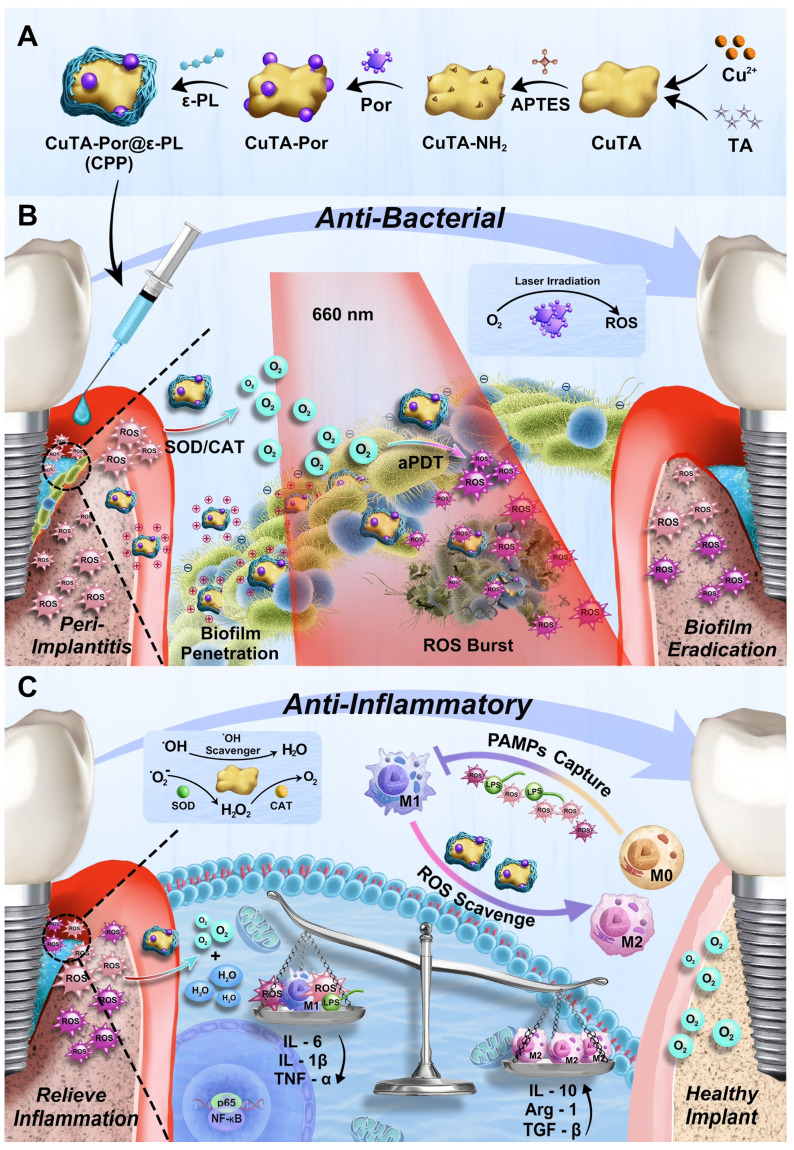
(A) Synthesis route of CPP NPs. (B) Antibacterial effect of CPP NPs. After synthesis, CPP NPs were injected into the peri-implant pocket, targeting and penetrating the biofilm through their favorable hydrated particle size and electrostatic interactions. High level of ROS in PI tissue can be converted by CPP NPs via SOD/CAT activity to generate oxygen, ameliorating the hypoxic condition of PI. The biofilm was then eradicated via aPDT upon 660 nm laser irradiation. (C) Anti-inflammatory effect of CPP NPs. After the biofilm eradication through ROS burst, CPP NPs still retain most of SOD/CAT activity, thereby rapidly clearing the residual ROS generated by aPDT. Additionally, by PAMPs blocking and endogenous ROS scavenging, the regulation of macrophage polarization was achieved. By regulating the generation and elimination of ROS from a temporal and spatial perspective, CPP NPs ultimately and effectively enhanced the treatment efficacy of PI.

In our study, as shown in Scheme 1A, we first synthesized and aminated CuTA NSs, then grafted Por onto CuTA - NH₂ to form CuTA - Por NSs, and finally modified CuTA-Por with ε-PL to obtain water-soluble, positively charged CuTA-Por@ε-PL nanoplatforms (CPP NPs). Similar to clinical treatment, CPP NPs are locally injected into the peri-implant pocket (Scheme 1B). Their excellent hydrated particle size and positive charge improve penetration in biofilms and enable bacteria-targeting. In PI tissue, CPP NPs convert high-level ROS to oxygen via SOD/CAT activity, relieving hypoxia. Through laser irradiation, CPP NPs enhance ROS burst in aPDT to eradicate biofilms, and still retain SOD/CAT activity post-irradiation to clear residual ROS and generate oxygen, which inhibits anaerobic bacteria. Additionally, CPP NPs can achieve free bacteria capture and pathogen-associated molecular patterns (PAMPs) blocking through electrostatic interaction, thus preventing bacterial recolonization and reducing the inflammatory response caused by PAMPs (Scheme 1C). In summary, CPP NPs were fabricated in a simple and economical way. By regulating the generation and elimination of ROS from a temporal and spatial perspective, CPP NPs successfully broke through the bottlenecks faced by aPDT, achieving both biofilm eradication and inflammation regulation. This provides a potential strategy for the clinical treatment of PI.

## Results and Discussion

### Synthesis and characterization of CPP NPs

**Figure 1:**
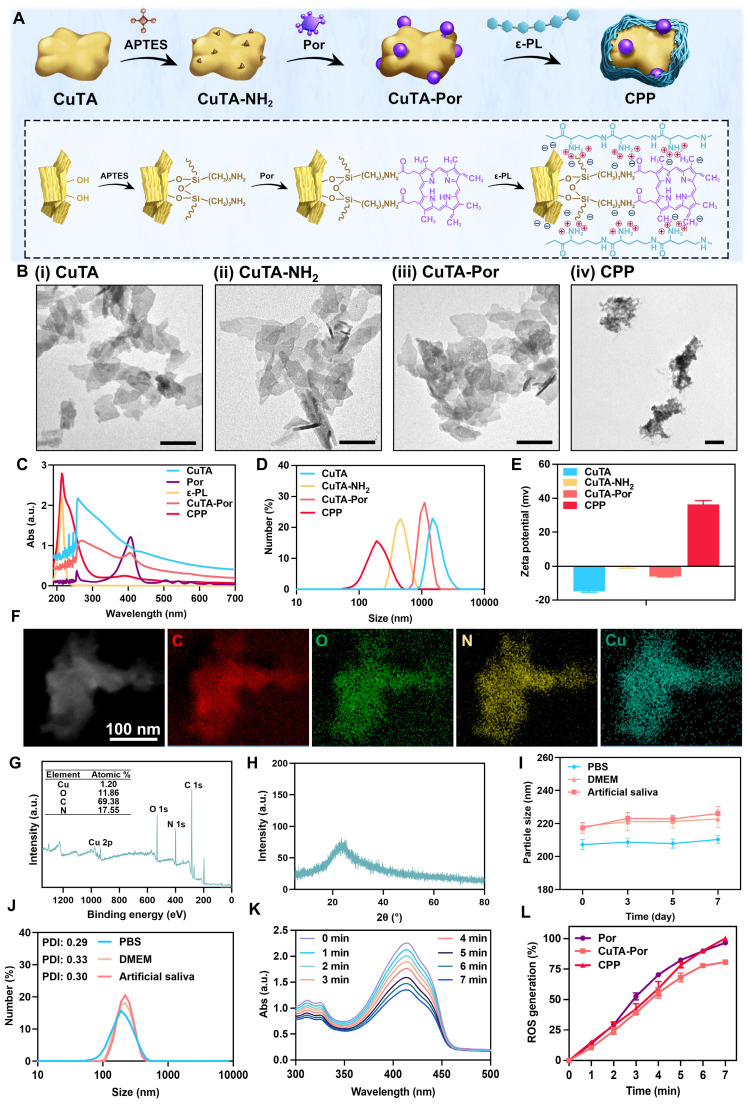
(A) Synthesis route of CPP NPs. (B) TEM images of (i) CuTA, (ii) CuTA-NH_2_, (iii) CuTA-Por, and (iv) CPP NPs. Scale bar: 100 nm. (C) UV-vis spectra of CuTA, Por, ε-PL, CuTA-Por, and CPP. (D) Hydrated particle size of CuTA, CuTA-NH_2_, CuTA-Por, and CPP. (E) Zeta potentials of CuTA, CuTA-NH_2_, CuTA-Por, and CPP. (F) SEM image and EDS-mapping analysis of CPP. (G) XPS spectrum of CPP. (H) XRD pattern of CPP. (I) The particle-size variation of CPP in different solutions over 7 days. (J) Size and PDI of CPP in different solutions after 7 days of incubation. (K) The absorption spectra of DPBF in CPP solution under different laser irradiation time (0-7 min). (L) Normalization analysis related to ROS generation of Por, CuTA-Por and CPP.

The synthetic route of CPP was illustrated in Figure 1A. First, the CuTA nanosheets (NSs) were synthesized following the method described in the literature 32, and then characterized by TEM, SEM, and infrared spectroscopy (Figure 1Bi, Figure S1A-B). The TEM and SEM results indicated that the product presented an excellent sheet-like structure. In the infrared spectrum, it was observed that compared with TA, the absorption characteristic peak centered at 3386 cm^-1^ was split. Meanwhile, the peaks in the fingerprint region of the CuTA NSs spectrum were shifted. This indicates that the coordination between the phenolic groups in TA and Cu^2+^ disrupts the C-OH vibration of TA. Both the morphology and infrared spectrum results confirmed the successful synthesis of CuTA nanosheets.

Next, APTES was used to modify the CuTA NSs so as to endow them with outward amino groups. Subsequently, the amino-modified CuTA (named CuTA-NH_2_) was covalently conjugated with the carboxyl groups of Por via EDC-NHS coupling, obtaining the formation of CuTA-Por NSs with PDT function. TEM images showed that the morphologies of CuTA, CuTA-NH_2_, and CuTA-Por had not undergone significant changes (Figure 1Bi-iii), suggesting that the treatment with APTES and the modification with Por had no obvious impact on the sheet-like structure of CuTA. After amino modification, the zeta potential of CuTA increased from -14.87 mV to -0.478 mV. Additionally, it further shifted to -6.04 mV after Por grafting (Figure 1E), confirming the successful modifications. After Por grafting, the color of the product transformed from green to purple (Figure S2). The ultraviolet spectrum results also indicated that CuTA-Por simultaneously exhibited the peak pattern of CuTA and the characteristic absorption band of Por at 410 nm, further confirming the successful synthesis of CuTA-Por nanosheets (Figure 1C).

High photodynamic performance is a prerequisite for exerting antimicrobial photodynamic therapy (aPDT). Thus, we synthesized CuTA-Por NSs with varying mass ratios of Por/CuTA (Figure S3C) and investigated *in vitro* ROS generation ability by using 1,3-diphenylisobenzofuran (DPBF) as a ROS probe (Figure S3Ai-iv, Figure S3B). The aPDT effect mainly relies on an instantaneous burst of ROS. The results in Figure S3B indicated that there was no significant difference in the photodynamic performance of CuTA-Por with a Por/CuTA mass ratio of 1:10 and 1:20 within the first five minutes (min). However, CuTA-Por with a Por/CuTA mass ratio of 1:40 and 1:60 showed insufficient ROS generation capacity within the first five min. Thus, we decided to select CuTA-Por (1:20) for the subsequent experiments. Unless otherwise specified in the following text, the CuTA-Por mentioned in this article refers to that with a Por/CuTA mass ratio of 1:20.

The TEM image (Figure 1Biii) revealed that CuTA-Por displayed a relatively small size (approximately 110 nm) in the dry state. However, it was observed that CuTA-Por had poor solubility in aqueous solutions and was prone to sedimentation. The hydrated particle size, as detected by DLS, was 1009 nm (Figure 1D). This characteristic is unfavorable for the penetration and dispersion of NPs within biofilms. To solve this problem, CuTA-Por was coated with ε-PL to prepare CPP NPs. After being coated with ε-PL, the solution transformed from a turbid purple (CuTA-Por) to a clear and translucent light green (CPP). Additionally, after centrifugation at 5000 rpm for 5 min, no precipitate was observed in the CPP solution (Figure S2). The coating of ε-PL not only improved the water solubility of CuTA-Por but also endowed the CPP NPs with a strong positive charge (Figure 1E). The favorable hydrated particle size (204.6 nm, Figure 1D) and positive surface charge (+36.4 mv) render CPP NPs more beneficial for deeply penetrating and dispersing within biofilms.

Furthermore, the ratio of CuTA-Por/ε-PL was adjusted to synthesize three types of CPP NPs with mass ratios of 1:5, 1:10, and 1:20 (Figure S3D). The results of DLS showed that the particle size of CPP (1:5) was mainly distributed within the range of 255 nm to 955 nm (PDI 0.690). The particle size of CPP (1:10) was 204.6 nm with a PDI of 0.291. The particle size of CPP (1:20) was 631.5 nm (PDI 0.470), and a peak of a larger-sized product appeared at 5000 nm. Based on the above results, it is considered that when the ratio was 1:5, CuTA-Por was difficult to be fully encapsulated, resulting in a relatively wide particle size distribution range. When the ratio was 1:20, excessive ε-PL caused some CuTA-Por to be overly thickly coated or aggregated, thereby forming products with larger particle sizes. When the ratio was 1:10, the product displayed an appropriate particle size and uniform distribution. Therefore, the ratio of 1:10 was selected as the optimal CuTA-Por/ε-PL ratio for CPP synthesis.

After synthesis, CPP was characterized by EDS mapping, X-ray photoelectron spectroscopy (XPS) and X-ray diffraction (XRD) patterns. The EDS mapping results of CPP indicate that the NPs contain C, O, N, and Cu elements (Figure 1F). In contrast to CuTA, the presence of the N element in CPP is notable (Figure S1A). The N element has three primary sources: the amination of CuTA with APTES; the amide bonds formed during the grafting of CuTA-NH_2_ with Por; and the presence of N within ε-PL. This was further corroborated by the XPS results (Figure 1G, Figure S3E-F). The XPS spectrum of CuTA did not exhibit any N-element signature, whereas the atomic percentage of N in CuTA-Por was 3.89%, confirming the successful introduction of amino/amide bonds. Following the encapsulation with ε-PL, due to the high N-content in ε-PL, the N-element content in CPP increased significantly, with an atomic percentage of 17.55%, thus validating the successful encapsulation of ε-PL. The XRD patterns reveal that upon grafting Por onto CuTA, the overall peak shape remains largely unaltered, yet the intensities of diffraction peaks at specific 2θ angles, such as 22.98° and 35.6°, have changed (Figure S3 G-H). After the encapsulation with ε-PL, the XRD pattern displays only a broad diffraction-peak envelope, devoid of sharp and discrete diffraction peaks (Figure 1H). This strongly suggested that CPP likely possessed an amorphous or non-crystalline structure, as opposed to a highly ordered crystalline one.

Subsequently, the particle size stability and dispersibility of CPP were measured. PBS, DMEM (with 10% FBS), and artificial saliva were chosen to do the test. The results indicated that the particle sizes of CPP in the three solutions did not change significantly within 7 days, confirming its good stability (Figure 1I). The slight increase in DMEM and artificial saliva could be attributed to protein adsorption. On the 7th day, we measured the particle size distribution and PDI of CPP in different solutions (Figure 1J). PDI is an important parameter used to measure the uniformity of the particle size distribution of nanoparticles. It reflects the degree of dispersion of particle sizes in the nanoparticle system. On the 7th day, the PDI values of CPP in the three solutions were all around 0.3, indicating that CPP had relatively good dispersibility.

In addition, the release of Cu^2+^ from CPP NPs was also investigated (Figure S3 I-J). The experimental results demonstrated that a small amount of Cu^2+^ was gradually released as time elapsed. This phenomenon might contribute to the antibacterial activities and the facilitation of tissue repair processes. Under acidic conditions, the quantity of Cu^2+^ released from CPP NPs was marginally higher than that under neutral and alkaline conditions. Next, CCK-8 and hemolysis assays were employed to validate the favorable biocompatibility of CPP NPs (Figure S3K-L). Remarkably, even at a concentration as high as 800 μg/mL, more than 80% of the HGFs and L929 cells maintained their viability, and the hemolysis rate was lower than 5%.

### ROS leverage based on CPP NPs

aPDT achieves bacterial killing through the generation of ROS. Nevertheless, ROS production lacks bacterial selectivity, and a high level of ROS can also trigger an endogenous inflammatory response, consequently leading to the production of more intracellular ROS in tissues 33. PI entails the destruction of both hard and soft tissues due to an excessive inflammatory response of the organism caused by plaque biofilm infection 10. After aPDT, the accumulated ROS aggravate the imbalance of oxidant/antioxidant activity in PI pockets, recruit inflammatory cells, and prompt the polarization of macrophages to the M1 phenotype, thereby further aggravating the destruction of hard and soft tissues. Therefore, when contemplating the use of aPDT to eliminate bacteria, the side effects of excessive oxidative stress that aPDT imposes on PI tissues should also be considered. Studying the generation, activity and reduction of ROS in terms of time dynamics is essential for resolving the contradiction between bactericidal treatment and inflammation.

#### ROS generation under laser irradiation

The transient burst of ROS induced by excitation light constitutes the main mechanism through which aPDT exerts its antibacterial effects 24. Under the irradiation of excitation light, PSs can instantaneously generate a large amount of ROS through photocatalysis. Therefore, exploring the impacts of CuTA and ε-PL encapsulation on the ROS generation performance of Por under laser irradiation should be given top priority.

DPBF was used to evaluate the PDT performances of CPP, CuTA-Por, and Por by the similar method described previously (Figure 1K-L). The results revealed that compared with Por, the PDT performance of CPP did not change significantly, which is of great significance for CPP NPs to achieve aPDT through a transient ROS burst within a short period. However, the PDT effect of CuTA-Por in water decreased due to its poor solubility. Therefore, the coating of ε-PL not only contributes to enhance the penetration and distribution within bacterial biofilms through its positive charge and small size, but also improves the PDT effect of CuTA-Por and further enhances the ROS burst.

#### ROS scavenging without laser irradiation

**Figure 2:**
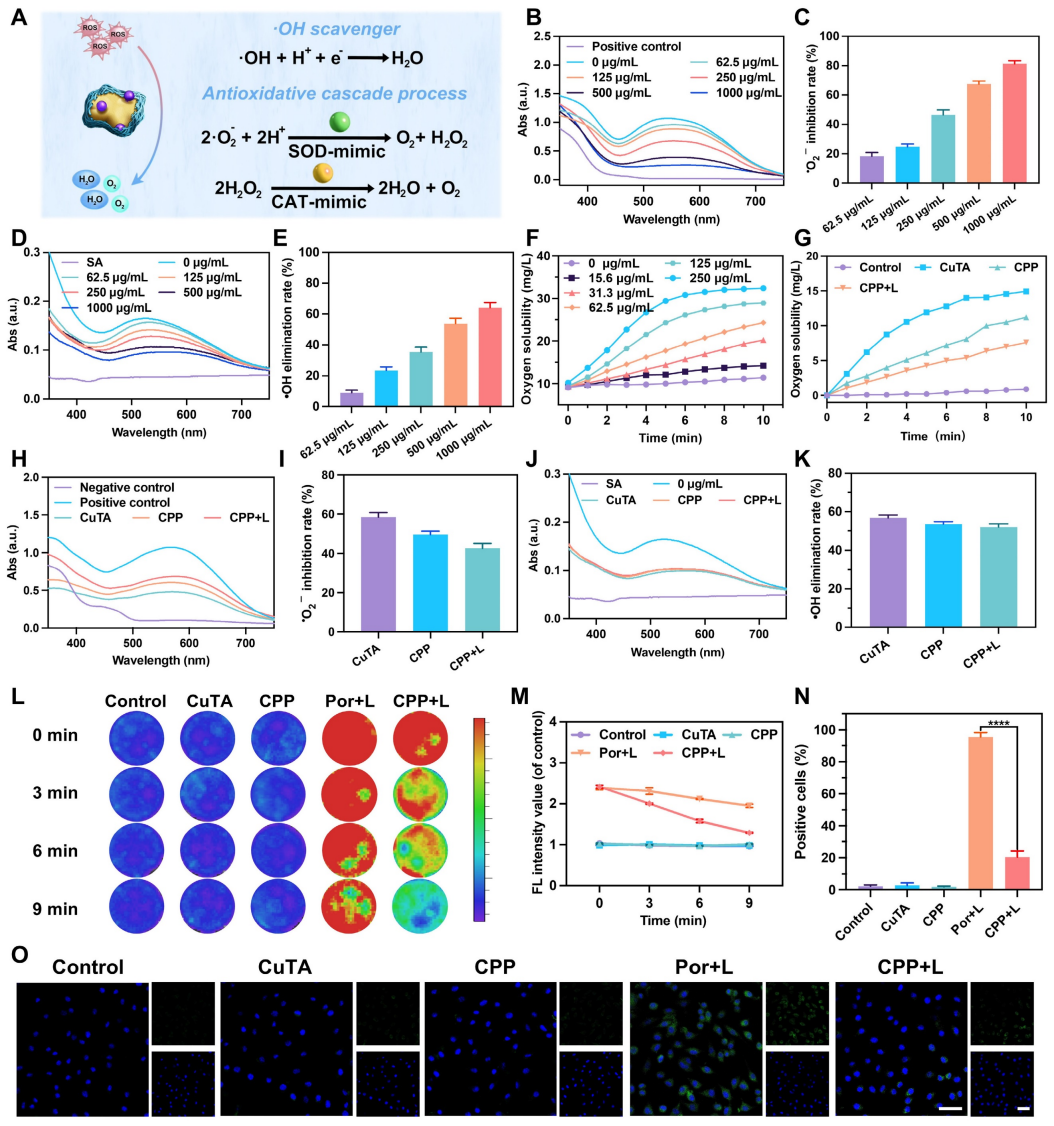
(A) Schematic illustration of the antioxidative activities of CPP NPs. (B) Absorption spectra of NBT reduced by **^•^**O^2-^ in the presence of varying concentrations of CPP NPs and (C) the associated normalization analysis. (D) Absorption spectra of SA reacted with **^•^**OH in the case of different concentrations of CPP NPs and (E) the related normalization analysis. (F) The concentrations of dissolved oxygen catalyzed by diverse concentrations of CPP NPs and (G) different solutions. (H) Absorption spectra of NBT reduced by **^•^**O^2-^ with different solutions and (I) the corresponding normalization analysis. (J) Absorption spectra of SA reacted with **^•^**OH with different solutions and (K) the relevant normalization analysis. (L) General *in vitro* fluorescence images of ROS generation and scavenging upon different treatments using the IVIS^®^ Imaging System, and (M) the corresponding fluorescence intensity analysis. (N) Quantification of the intracellular ROS level in HGFs after different treatments and (O) CLSM images (scale bar: 50 μm). Data are shown as mean ± SD (n = 3) (****P < 0.0001).

The main mechanism of CPP NPs to scavenge ROS stems from the SOD/CAT enzyme activity of CuTA (Figure 2A). To mitigate the side effects induced by aPDT, a crucial problem requires verification, specifically that CPP NPs possess SOD/CAT enzyme activity and that this activity remains even after laser irradiation. Only in this way can the ROS leveraging mode of “generating ROS for antibacterial effect under laser irradiation and scavenging ROS for anti-inflammatory effect without laser irradiation” be achieved.

To examine the inherent SOD-like activity of CPP NPs, the dismutation of superoxide radicals catalyzed by CPP NPs was determined by measuring the inhibition ratios of the photoreduction of nitro blue tetrazolium (NBT) 32. The superoxide anions (**^•^**O₂⁻) were produced by riboflavin and methionine in PBS (pH 7.4) after ultraviolet irradiation. NBT, a sensitive superoxide indicator, forms blue methyl hydrazone with an absorption at 560 nm upon reduction by **^•^**O₂⁻. As illustrated in Figure 2B, the absorbance value at 560 nm significantly decreased in the presence of CPP at different concentrations. At a CPP concentration of 250 μg/mL, approximately 50% of **^•^**O₂⁻ could be inhibited (Figure 2C). In addition, the hydroxyl radicals (**^•^**OH) elimination efficiency of CPP NPs was ascertained by measuring salicylic acid (SA). SA can be oxidized and consumed by **^•^**OH, thereby generating 2,3-dihydroxybenzoic acid with a purple color and an absorption peak at 510 nm. As depicted in Figure 2D, the intensity of the absorption peak at 510 nm diminished in the presence of CPP NPs. The **^•^**OH elimination efficiency exhibited a concentration-dependent manner. More than 50% of **^•^**OH could be eliminated when CPP NPs at a concentration of 500 μg/mL were present, indicating an excellent **^•^**OH scavenging capacity (Figure 2E).

To demonstrate the CAT-like activity of CPP NPs, the decomposition performance of hydrogen peroxide (H_2_O_2_) was evaluated. Upon adding CPP NPs to PBS (0.1 M, pH 7.4) containing H_2_O_2_, bubbles could be observed, and the number of bubbles increased in proportion to concentrations of CPP NPs (Figure S4). To clarify this phenomenon, the generation of oxygen was further monitored with a dissolved oxygen meter. As depicted in Figure 2F, the decomposition rates increased in accordance with the concentration of CPP NPs, indicating the inherent CAT-like activity of CPP NPs.

In conclusion, CPP NPs exhibited satisfactory scavenging capacity for various ROS by simulating the SOD and CAT cascade process, catalyzing the dismutation of **^•^**O_2_⁻ to H_2_O_2_, and then converting H_2_O_2_ into O_2_ and H_2_O. PI tissues are in a state of excessive oxidative stress. The SOD/CAT activity of CPP NPs is beneficial for converting ROS in PI pockets into oxygen and water. The generated O_2_ is advantageous for improving the hypoxic condition in PI pockets, thus enhancing the aPDT effect produced by subsequent laser irradiation.

Subsequently, we used the same approach as described above to compare the ROS scavenging ability of CPP NPs before and after irradiation. The results indicated that the ability of CPP NPs to scavenge **^•^**O_2_⁻ (Figure 2H-I) and **^•^**OH (Figure 2J-K) did not exhibit significant changes after 5 min of 660 nm laser irradiation, and the ability to scavenge H_2_O_2_ was still retained at 67.8% (Figure 2G). Consequently, after CPP NPs exert their aPDT function, they can still perform the ROS scavenging function via SOD/CAT enzyme activity, thus avoiding the excessive oxidative stress side effects of aPDT on tissues and continuously reducing the ROS level.

#### Dynamic monitoring of the generation and scavenging of ROS* in vitro*

To achieve a more direct and intuitive visualization of the generation and scavenging of ROS 21, 24, five groups of stock solutions were prepared separately. The Por+L and CPP+L groups were exposed to 660 nm laser irradiation for 5 min, while the other groups remained untreated. Subsequently, 180 μL of the solution was promptly extracted from the stock solution and added to the well plate, followed by the addition of DCFH-DA. The fluorescence imaging was instantaneously recorded on the IVIS^®^ Imaging System (λex = 488 nm, λem = 525 nm), and this was designated as “0 min” (Figure 2L). Subsequently, every 3 min, 180 μL of the solution was withdrawn from the stock solution, added to the well plate, and DCFH-DA was added, and the previously described detection process was repeated. The results demonstrated that after irradiation, the fluorescence intensities of the Por+L and CPP+L groups increased significantly and were comparable in magnitude, thus confirming the ROS generating capability of CPP NPs under laser irradiation. After the termination of laser irradiation, the fluorescence intensity of CPP+L group decreased continuously, and 78.1% of the ROS had been eliminated by 9 min (Figure 2M), thereby verifying the ROS scavenging ability of CPP NPs in the absence of laser irradiation.

Using the same grouping method, the generation and elimination of intracellular ROS were detected. As shown in Figure 2N-O, in the Por+L group, the proportion of positive cells was as high as 95.6%, indicating that aPDT treatment could significantly cause oxidative stress in cells. While in the CPP+L group, this proportion decreased to only 20.35%, demonstrating that CPP NPs effectively alleviate the intracellular high ROS state caused by aPDT.

### The strong biofilm penetration and preferential bacterial selectivity mediated by CPP NPs

Bacteria usually present a negative charge on their surfaces 34, 35. For Gram-positive bacteria, their cell walls are primarily composed of peptidoglycan and teichoic acid. Under physiological conditions, the phosphate groups within teichoic acid will ionize, releasing hydrogen ions and thereby acquiring a negative charge 36. In the case of Gram-negative bacteria, their outer membranes contain a large quantity of lipopolysaccharide. The phosphate and carboxyl groups on the lipopolysaccharide are more prone to dissociation in the physiological environment, generating a substantial amount of negative charges. This results in the outer potential of Gram-negative bacteria cells being more negative than that of Gram-positive bacteria 37.

**Figure 3:**
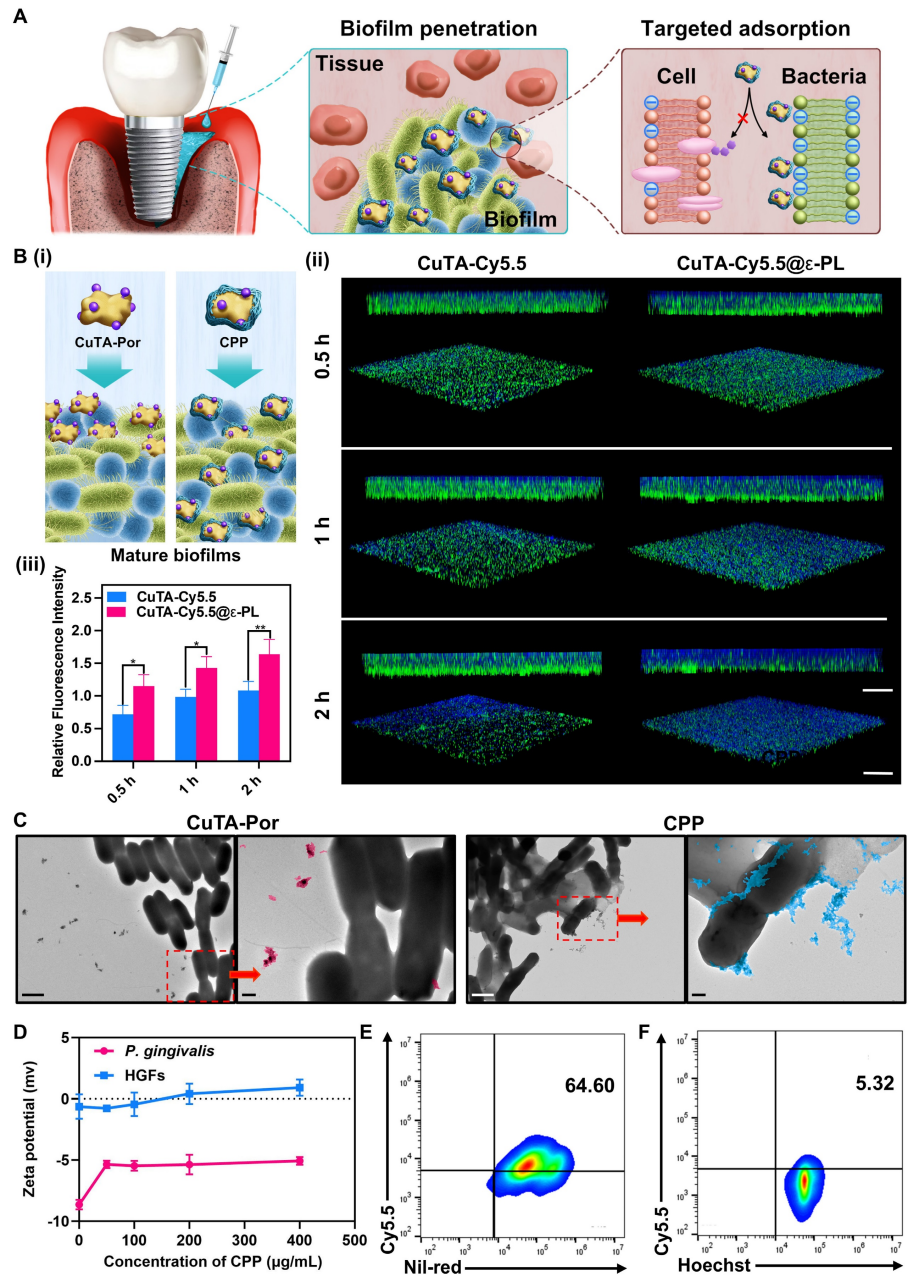
(A) Scheme of biofilm penetration and selective adsorption mediated by CPP NPs. (B) (i) Scheme of biofilm penetration mediated by CuTA-Por and CPP NPs. (ii) 3D CLSM images (scale bar: 100 μm) and corresponding z-stack images (scale bar: 20 μm) of *P. gingivalis* biofilms treated with CuTA-Cy5.5 and CCP for different time, and (iii) relative fluorescence intensity. (C) TEM images of *P. gingivalis* after treatment with CuTA-Por and CPP (scale bar: 1 μm), as well as the local enlargements of these images (scale bar: 200 nm). (D) Zeta potential of *P. gingivalis* and HGFs cells after mixing with CPP NPs at different concentrations for 30 min. (E) Flow cytometry analysis of co-cultured *P. gingivalis* (stained by Nile-Red) and (F) HGFs cells (stained by Hoechst 33342) after adding CPP NPs for 30 min. Data are shown as mean ± SD (n = 3) (*P < 0.05 and **P < 0.01).

CPP NPs possess a highly positively-charged surface. Hence, we hypothesized that CPP NPs can interact strongly with the typical pathogen of PI (*P. gingivalis*), thereby enhancing the penetration of biofilms. Moreover, the surfaces of mammalian cells are usually electrically neutral 33. This distinct surface property compared to bacteria might enable CPP NPs to bind preferentially to bacteria more easily, thus strengthening the selectivity for bacteria (Figure 3A).

#### Biofilm penetration and dispersion mediated by CPP NPs

Bacterial biofilms are complex structures formed by bacteria and the extracellular polymers they secrete 38. The biofilms structure significantly affects the distribution of PSs. Specifically, it causes a relatively low concentration of PSs within the biofilm. Additionally, the ROS generated by aPDT on the biofilm surface has insufficient penetrating ability, making it difficult for them to reach the biofilm interior. Hence, implementing measures to enhance the penetration and dispersion of PSs is an effective approach to achieve complete biofilms eradication and overcome the limitations of aPDT.

To investigate the penetration and accumulation of CPP NPs in mature biofilms, CuTA-Cy5.5 and CuTA-Cy5.5@ε-PL(CCP) were synthesized for simulating CuTA-Por and CPP NPs (Figure 3Bi). After constructing of mature *P. gingivalis* biofilms, CuTA-Cy5.5 and CCP NPs were respectively co-incubated with the biofilms for 0.5, 1, and 2 h. Subsequently, unbound NPs were washed off, and the biofilms were labeled with DMAO and observed via CLSM (Figure 3Bii-iii). The results indicated that in both the CCP and CuTA-Cy5.5 groups, the penetration depth and NPs accumulation increased over time. Nevertheless, the penetration and distribution in the CuTA-Cy5.5 group were evidently non-uniform, which could be ascribed to the large hydrated particle size of CuTA-Por (1009 nm). Although the TEM images of CuTA-Por revealed a sheet-like structure of approximately 110 nm, it exhibits poor water solubility, leading to insufficient penetration depth and uneven distribution in the biofilm. After being coated with ε-PL, the hydrated particle size of CPP was sharply reduced to 204.6 nm, and the surface showed a strong positive charge of +36.4 mv. This greatly enhanced the penetration ability of CPP NPs into the biofilm. After 2 h of co-incubation, the CCP group showed a uniform blue fluorescence distribution, and the penetration nearly reached the entire layer of the biofilm. The fluorescence intensity was 1.5 times that of the CuTA-Cy5.5 group, demonstrating that the construction strategy of CPP NPs can effectively solve the problem of insufficient PSs penetration.

#### Selective absorption of CPP NPs on cells and bacteria

The positive charge of CPP NPs plays a crucial dual role. First, it enhances the penetration and dispersion of CPP NPs within biofilms. This characteristic enables the NPs to reach their target sites within the biofilm environment more efficiently. Secondly, it facilitates the preferential interaction of CPP NPs with bacteria. This specific interaction is important as it enables the NPs to act on the bacteria more precisely and also mitigates potential side effects on surrounding tissues 39. PI is mainly caused by pathogenic bacteria such as *P. gingivalis* and* F. nucleatum*, which are Gram-negative bacteria. The cell walls of these bacteria are rich in lipopolysaccharide, endowing them with a negative surface charge. In Figure 3C, a conspicuous interaction between CPP NPs and *P. gingivalis* was observed. The CPP NPs either penetrated into the bacterial interior or adhered to the bacterial surface, whereas the electronegative CuTA-Por had only a limited amount of binding and the majority remained unbound outside the bacteria. This difference highlights the significance of the positive charge feature of CPP NPs in their selective interaction with bacteria.

Most cell membranes of mammals are electrically neutral 33. This essential property forms the foundation for inferring that positively charged NPs will preferentially bind to negatively charged bacteria based on electrostatic interaction principles. To validate this hypothesis, we co-incubated different concentrations of CPP NPs with *P. gingivalis* for 30 min. Subsequently, the bacteria were collected by centrifugation, unbound NPs were washed away, and then the bacteria were resuspended and their surface charge was measured (Figure 3D). The results indicated that as the concentration of CPP NPs increased, the surface charge of the bacteria gradually rose from -8.6 mv initially to -5.06 mv. This upward trend demonstrated that CPP NPs have a strong interaction with bacteria. Using a similar approach, the surface potential of HGFs after incubation with CPP NPs was determined. The results demonstrated that the Zeta potential of HGFs only increased from -0.63 mV to +0.9 mV. This relatively minor change in potential indicated that the interaction between CPP NPs and HGFs cells was relatively weak.

To further investigate the preferential selectivity of CPP NPs for bacteria over cells, Hoest 33342-labeled HGFs and Nile red-labeled *P. gingivalis* were mixed and then co-incubated with CPP NPs for 30 min. Subsequently, flow cytometry was employed to explore the uptake of CPP NPs in the presence of both bacteria and cells (Figure 3E-F). The results demonstrated that the binding ratio of CPP NPs to *P. gingivalis* was approximately 12.1 times that to HGFs, indicating a significant preference for binding to bacteria rather than mammalian cells.

Based on the aforementioned data, CPP NPs can decrease their accumulation in cells by virtue of their preferential binding selectivity towards bacteria. This unique property not only enhances their antibacterial capacity but also effectively reduces their potential adverse effects on cells.

### Antibacterial and anti-biofilm properties of CPP NPs

PI presents significant challenges in clinical practice, primarily due to the intricate processes of bacterial colonization and biofilm formation. Biofilms, particularly those associated with *P. gingivalis*, occupy a central position in the pathogenesis of PI 40. Mature biofilms can initiate a complex cascade of immune responses, thereby causing significant damage to the tissues surrounding implants.

Effectively managing biofilms is a cornerstone in PI treatment. This entails not only the complete elimination of existing mature biofilms but also the strict prevention of their re-formation. Additionally, the free bacteria released from mature biofilms complicate the situation further. These free-floating bacteria can migrate and recolonize in other areas, thereby generating new biofilms. Moreover, they can infiltrate adjacent tissues, exacerbating the inflammatory process within the peri-implant environment 41.

Consequently, there is an urgent and crucial demand for innovative therapeutic agents capable of simultaneously targeting both free bacteria and biofilms. In this study, we focused on investigating the inhibitory effects of CPP NPs on free bacteria, mature biofilms, and biofilm formation processes. From multiple aspects, we comprehensively demonstrated the excellent antibacterial and antibiofilm capabilities of CPP NPs and elaborated on their connection with effective PI treatment.

#### Capturing and killing of free bacteria

Initially, the plate colony counting approach was employed to investigate the antibacterial efficacy of different concentrations of CPP NPs against free *P. gingivalis* (after 5 min laser irradiation and 24 h co-incubation) within 24 h. The results showed that when the concentration of CPP NPs was only 200 μg/mL, more than 60% of the bacteria could be eliminated. When the CPP concentration reached 400 μg/mL, free bacteria could be completely eradicated (Figure S5Ai, Figure S5B). Moreover, TEM images also showed that as the concentration increased, more CPP NPs interacted with the bacterial cell membrane or penetrated into the interior of the bacteria, and the bacterial morphology gradually disintegrated (Figure S5Aii).

Subsequently, with the concentration of CPP NPs fixed at 300 μg/mL, five groups (control, CuTA, Por+L, CPP, and CPP+L group) with or without laser irradiation were established to further investigate the antibacterial efficacy of CPP NPs. The results demonstrated that the antibacterial effect of CPP group was 35.3% higher than that of CuTA group (Figure S5C, Figure S5Fi). This might be attributed to the positive charge of CPP NPs enhancing its interaction with bacteria. The antibacterial effect of the CPP+L group was superior to that of the CPP group, confirming that CPP NPs can effectively kill bacteria through the PDT effect. Furthermore, the morphological changes of bacteria in each group after treatment were observed via TEM (Figure S5Fii). The images revealed that all groups except the control group exhibited damage and disintegration of the bacterial cells. However, significant agglomeration of bacteria was observed in the CPP and CPP+L groups. It can be seen that CPP NPs are distributed both on the bacterial membrane and inside the bacteria, which allows CPP NPs to adsorb bacteria together to form bacterial clusters through interaction while killing bacteria. This phenomenon was also manifested after the co-incubation. It could be observed that there was no significant precipitation in the Control, Por, and CuTA groups. However, the dark precipitate appeared in the CPP and CPP+L groups, especially in the CPP+L group (Figure S5Fiii). This was caused by the interaction between CPP NPs and bacteria, which led to the formation of larger aggregates and consequently resulted in sedimentation. Additionally, the more conspicuous bacterial sedimentation in the CPP+L group was ascribed to the greater number of bacteria killed by aPDT-mediated ROS burst. After maturation, biofilms will release free bacteria. These free bacteria can recolonize at other locations to form biofilms or further spread to the tissues surrounding implants, thereby exacerbating tissue damage 42. The ability of CPP NPs to capture and kill free bacteria may block this process and thus enhance the treatment efficacy.

In the TEM images (Figure S5Aii, Figure S5Fii), the rupture of the bacterial cell membrane can be clearly observed. To further assess the destruction of the cell membrane, a nucleic acid leakage experiment was employed. The destruction of the cell membrane leads to the release of intracellular components such as DNA and RNA into the surrounding environment, ultimately culminating in bacterial death. As depicted in Figure S5D, in the CPP+L group, the value of OD260 significantly increased after laser irradiation, and it still exhibited an upward trend even after the irradiation stopped. This phenomenon was also observed in the protein leakage detection (Figure S5E), confirming that in addition to interacting with bacteria and causing membrane damage, the burst of ROS generated by CPP NPs upon laser irradiation can also cause significant rupture to bacteria. In addition, we investigated the antibacterial effect of CPP after incubation in PBS for 7 days to explore the stability of its antibacterial properties. The results showed that there was no significant difference in the antibacterial activity of CPP against *Staphylococcus aureus* (*S. aureus*) (Figure S6A, Figure S6C) and *Escherichia coli* (*E. coli*) (Figure S6B, Figure S6D) after 7-day incubation compared with that of non-incubated CPP, which confirmed that the antibacterial performance of CPP was relatively stable.

#### Eliminating mature biofilms and inhibiting biofilm formation

**Figure 4:**
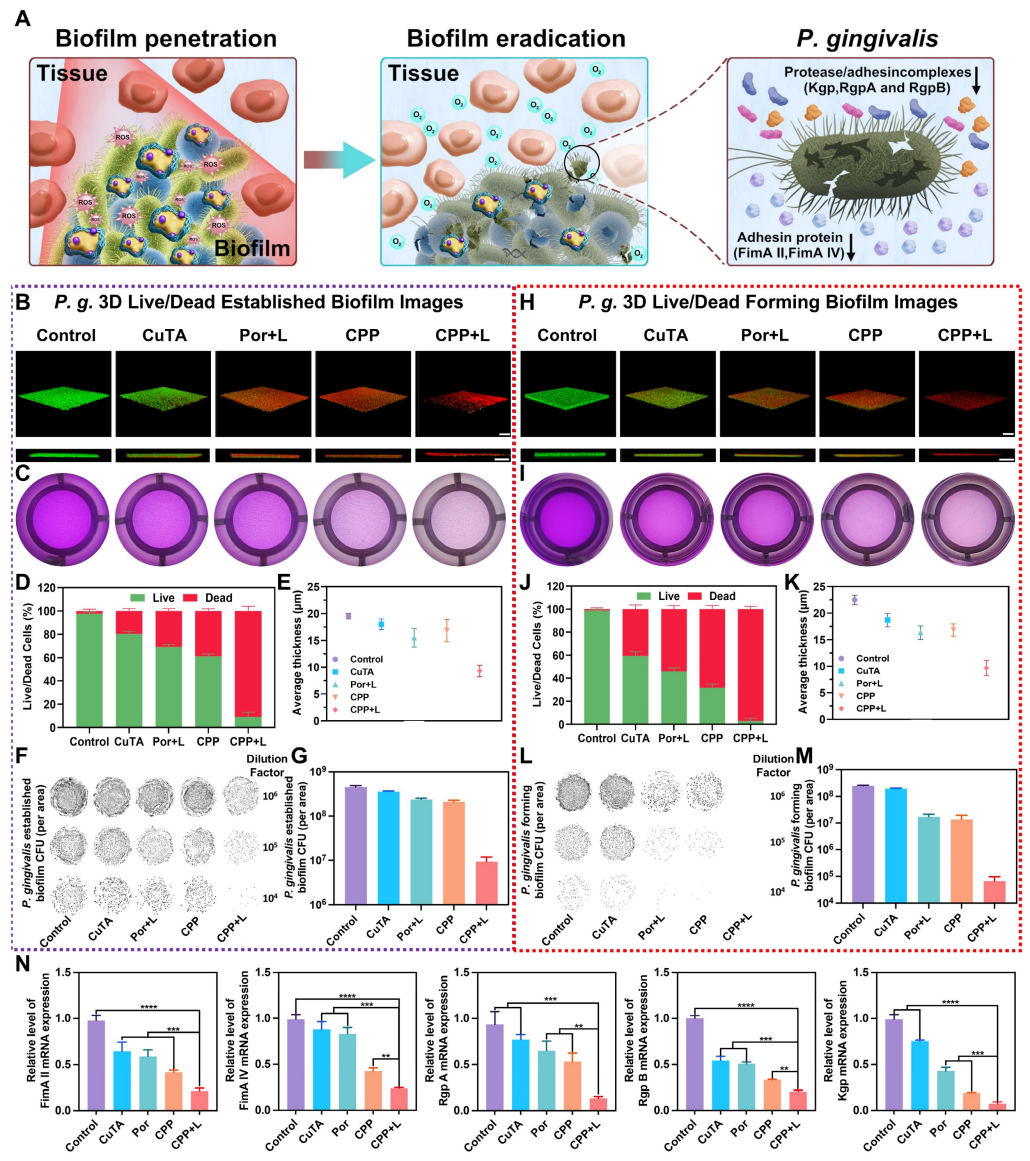
Antibacterial actions on the established and forming biofilms of *P. gingivalis*. (A) Diagram of the anti-biofilm function of CPP NPs. (B) Representative 3D Live/Dead pictures of the established biofilms of *P. gingivalis*. Scale bar: 100 μm. (C) Representative images of the established biofilm after crystal violet staining. (D) Proportion of dead to live bacteria for *P. gingivalis* in the established biofilm. (E) Mean thickness of the established biofilms of *P. gingivalis*. (F) Representative photos of bacterial colonies of the established biofilms of *P. gingivalis*, and (G) relevant quantitative analysis. (H) Representative 3D Live/Dead pictures of the forming biofilms of *P. gingivalis*. Scale bar: 100 μm. (I) Representative images of the forming biofilm after crystal violet staining. (J) Proportion of dead to live bacteria for *P. gingivalis* in the forming biofilm. (K) Mean thickness of the forming biofilms of *P. gingivalis*. (L) Representative photos of bacterial colonies of the forming biofilms of *P. gingivalis*, and (M) relevant quantitative analysis. (N) RT-qPCR examination of the relative mRNA levels of adhesin molecule genes and virulence factor genes in the established *P. gingivalis* biofilms. Data are shown as mean ± SD (n = 3) (**P < 0.01, ***P < 0.001, and ****P < 0.0001).

The foremost problem in treating PI is the efficient elimination of plaque biofilms. In this research, CPP NPs exhibited outstanding biofilm elimination capabilities (Figure 4A). As shown in Figure 4B-E and Figure S7A, the Por+L group demonstrated a certain degree of anti-biofilm effect. However, there were still more than 60% of viable bacteria remaining, and the thickness of the biofilm only showed a slight reduction. In contrast, compared with the Por+L group, the anti-biofilm effect of the CPP+L group was increased by 2.95 times, and the biofilm thickness was only 55.2% of that of the Por+L group. This validated that CPP NPs could effectively improve the efficacy of aPDT in anti-biofilm. The results of plate colony counting also confirmed that CPP NPs could effectively kill the bacteria within the biofilm (Figure 4F-G). Specifically, the number of bacteria in the CPP+L group decreased by 48.9 times in comparison with the control group. In addition, we constructed biofilms of *S. aureus* and* E. coli*, and treated them with the same method, which further verified the potential of CPP NPs in combating different bacterial biofilms (Figure S8).

*P. gingivalis* within biofilms is a crucial and typical pathogen of PI. It can generate multiple virulence factors to trigger the host's inflammatory response, thereby destroying the peri-implant tissues 43. Consequently, we investigated the inhibitory effect of CPP NPs on *P. gingivalis* by examining the expression of its virulence factors in biofilms after treatment with CPP NPs. Fimbriae (Fim) and gingipains are the key virulence factors of *P. gingivalis* 44. These factors are associated with biofilm formation and tissue destruction as they mediate the adhesion and aggregation of bacteria and facilitate the invasion of human epithelial cells. FimA genotypes II and IV show stronger pathogenicity than other fimA genotypes. Meanwhile, gingipains, which are the Arg-specific and Lys-specific proteinases of *P. gingivalis*, are encoded by RgpA, RgpB genes, and Kgp genes 45. In Figure 4N, the DNA levels of virulence factors in biofilms were presented. The results demonstrated that aPDT mediated by CPP NPs led to a significant reduction in the expression of *P. gingivalis* virulence factors. This reduction could be attributed to the fact that the production of ROS was capable of interacting with lipids and proteins. Subsequently, this interaction induced irreversible oxidative damage to DNA, RNA, and transcription factors, which in turn led to a decrease in virulence factor genes. Therefore, CPP NPs can disrupt the adhesion, aggregation, and parasitism processes of *P. gingivalis*, thus showing excellent therapeutic efficacy for PI.

In clinical practice, one of the challenges in the radical treatment of PI lies in the re-colonization and formation of biofilms 42. Therefore, inhibiting biofilm formation is equally crucial for the treatment of PI. Similarly, we investigated the inhibitory effect of CPP NPs on biofilm formation by means of live-dead staining of biofilms and plate colony counting (Figure 4H-M, Figure S7B). The results revealed that treatment with CPP NPs significantly decreased the thickness of the biofilm. The ratio of dead/live bacteria demonstrated that the ratios in groups treated with NPs were lower than those in mature biofilms, which was due to the incompletely formed structure of biofilm, leading to lower resistance to the treatment.

Overall, CPP NPs can capture and kill bacteria by causing damage to bacterial cell membranes and nucleic acids through strong interactions with bacteria and ROS burst, thereby achieving the removal of biofilms and inhibiting the formation of biofilms.

#### Transcriptomic analysis of *P. gingivalis* upon treatment of CPP NPs

**Figure 5:**
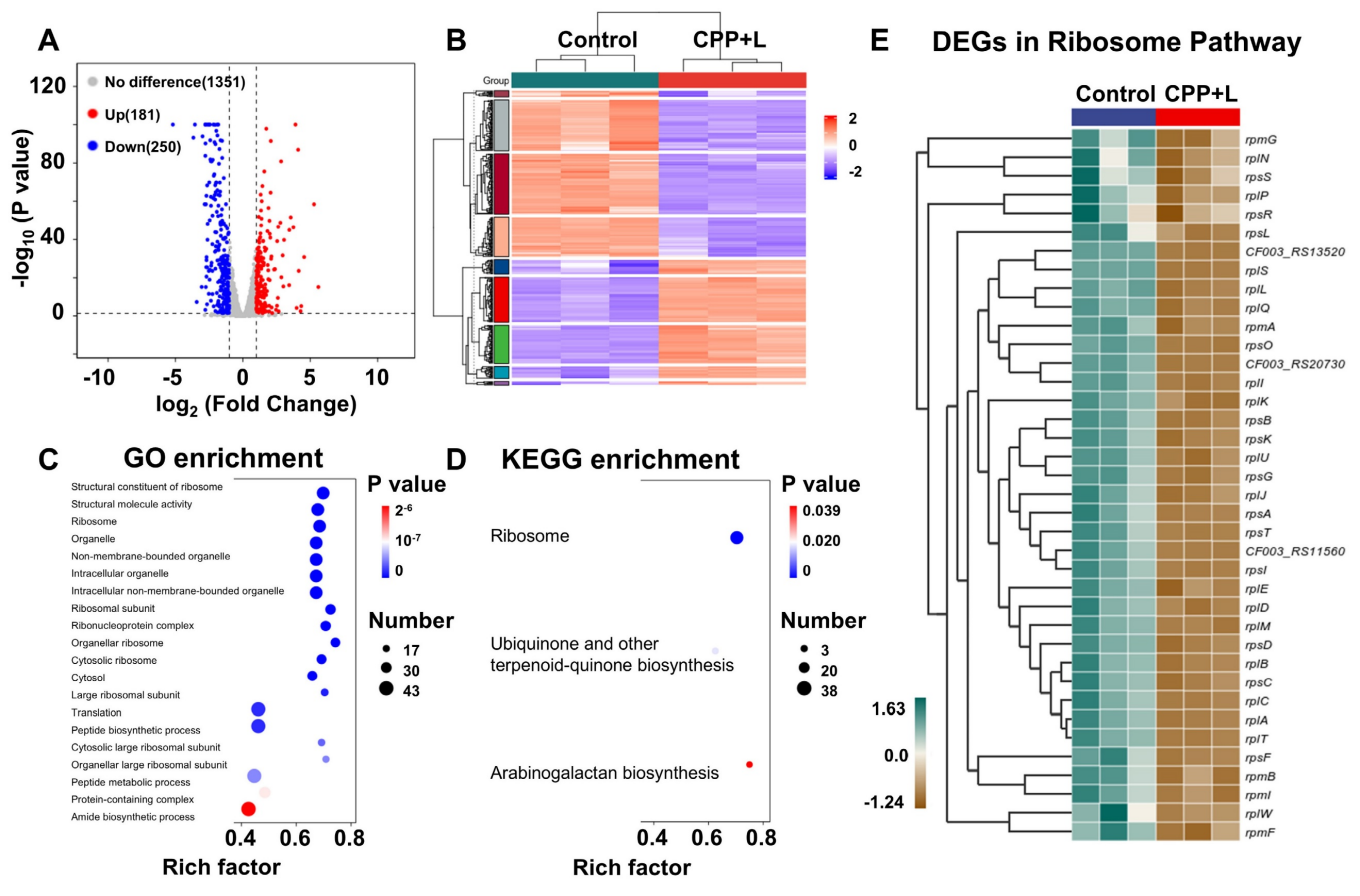
Global assessments of the* P. gingivalis* biofilm after the treatment with CPP NPs using RNA-seq. (A) Volcano plots of up-regulated and down-regulated genes in treated biofilm. (B) Heatmap of DEGs between Control and CPP+L group. (C) GO enrichment analysis of the DEGs. (D) KEGG enrichment analysis of the DEGs. (E) Interactive heatmap of significantly DEGs in Ribosome Pathway.

To further explore the antibacterial mechanism of CPP NPs, *P. gingivalis* was chosen as the model organism. A comparative transcriptomic analysis was carried out between the control group and the CPP+L group. As shown in Figure S9A, unsupervised principal component analysis demonstrated significant transcriptomic differences between the control and CPP+L groups. Based on the empirical Bayes method with strict criteria (fold change ≥1; p value <0.05), a total of 431 significantly differentially expressed genes (DEGs) were detected upon exposure to CPP NPs, including 250 downregulated genes and 181 upregulated genes. These results are visually presented in the volcano plots and heatmap (Figure 5A-B, Figure S9B). Furthermore, subsequent Gene Ontology (GO) and Kyoto Encyclopedia of Genes and Genomes (KEGG) enrichment pathway analysis indicated that after treatment with CPP+L NPs, ribosome damage was the most prominent (Figure 5C-D). This damage is attributed to the intense ROS burst mediated by CPP+L NPs.

Ribosomes serve as the sites for protein synthesis within cells. Regarding *P. gingivalis*, its survival and reproduction rely on the synthesis of a large amount of proteins. These proteins include enzymes involved in bacterial metabolic processes (such as those related to sugar metabolism and amino acid metabolism), proteins that constitute bacterial structures (such as components of cell walls and cell membranes), and virulence factors related to bacterial pathogenicity 46. For example, gingipain of *P. gingivalis* is an important virulence factor 44. It is a cysteine protease that can degrade host extracellular matrix proteins like collagen. The synthesis of gingipain requires ribosomes. If the function of ribosomes is damaged, the synthesis of gingipain will be inhibited. CPP+L NPs can affect *P. gingivalis* metabolism and structure by damaging bacterial ribosomes, thereby effectively controlling bacterial growth and reproduction and reducing bacterial pathogenicity. Additionally, damaging ribosomes starts from the basic life activity of bacteria-protein synthesis. Bacteria are less likely to develop drug resistance against this mechanism, which aligns line with the advantages of aPDT treatment. The significantly DEGs related to ribosomes after treatment with CPP+L NPs were listed in detail in the interactive heatmap of Figure 5E.

Furthermore, KEGG pathway analysis indicates that the treatment with CPP+L NPs simultaneously affects the biosynthesis of arabinogalactan, ubiquinone and other terpenoid quinones (Figure 5D). Arabinogalactan serves as an essential component of the biofilm formed by *P. gingivalis*. During biofilm formation, arabinogalactan enables bacteria to adhere to one another and attach to regions such as the surface of teeth/implants and oral mucosa 47, 48. Following treatment with CPP+L NPs, the synthesis of arabinogalactan was inhibited, which impeded the formation of a complete and sturdy *P. gingivalis* biofilm, thereby reducing the colonization ability of bacteria around the implants. Ubiquinone and menaquinone are crucial electron carriers in the cellular respiration process. They can transfer electrons between respiratory chain complexes, facilitating the pumping of protons from the mitochondrial matrix (a cytoplasmic region similar to that in bacteria) to the intermembrane space, thereby forming a proton motive force. This proton motive force drives the synthesis of ATP, which provides energy for various life activities such as bacterial growth, reproduction, movement, and the production of virulence factors 49, 50. After treatment with CPP+L NPs, the synthesis of ubiquinone and menaquinone in *P. gingivalis* was obstructed, resulting in hampered energy production in bacteria and an inability to synthesize ATP normally. Without sufficient energy supply, the growth rate of *P. gingivalis* will be significantly reduced. Insufficient energy supply also leads to a decrease in the production and secretion of virulence factors, correspondingly weakening the ability to damage peri-implant tissues.

### Anti-inflammatory activities of CPP NPs against LPS and aPDT-induced inflammation

In the pathogenesis of PI, bacterial colonization activates the host immune system, leading to a cascade of inflammatory responses. This innate immunity recruits various immune cells, including macrophages, to eliminate exogenous pathogenic stimuli. During the innate immune response, macrophages can change phenotypes and secrete various cytokines to combat stimulants 51.

M1-type macrophages are mainly involved in defending against invading pathogens, secreting destructive proinflammatory mediators, and generating endogenous ROS. Inflammatory phenotype macrophages are activated by bacterial LPS stimulation 52. LPS can bind to the Toll-like receptor 4 (TLR4) of macrophages, subsequently activating nicotinamide adenine dinucleotide phosphate oxidase (NOX2), triggering respiratory burst, and forming superoxide anion radicals 53. The accumulation of ROS increases the oxidative stress in surrounding tissues, thereby exacerbating peri-implant inflammation and alveolar bone resorption. Consequently, the overreaction of the M1 macrophage-related response may accidentally damage the surrounding normal tissue, resulting in the destruction of the peri-implant tissue. Additionally, although traditional aPDT can kill the bacteria around implants, the large amount of ROS it generates will further aggravate the oxidative stress state of tissues 24, thereby exacerbating the damage to the surrounding tissues.

Studies have shown that M2-phenotype macrophages exhibit pro-resolving, anti-inflammatory, and pro-regenerative activities in promoting wound healing, osteogenesis, osseointegration, and secreting anti-inflammatory cytokines 54. PI is an unregulated inflammatory response that leads to the development of pathological fibrosis, disrupting normal tissue architecture and function and simultaneously hindering many aspects of tissue repair and regeneration. Thus, the ultimate objective of treating inflammation is to control the local inflammatory response and reactivate the regenerative capacity of injured tissue.

Overall, the regulation of macrophage polarization holds great significance in the treatment of PI. In this part, we have probed into the ability and specific mechanism of CPP NPs in regulating macrophage polarization and have conducted an in-depth exploration of their effect in counteracting the inflammatory response of PI.

#### Inhibition of M1 macrophage polarization induced by LPS and aPDT

**Figure 6:**
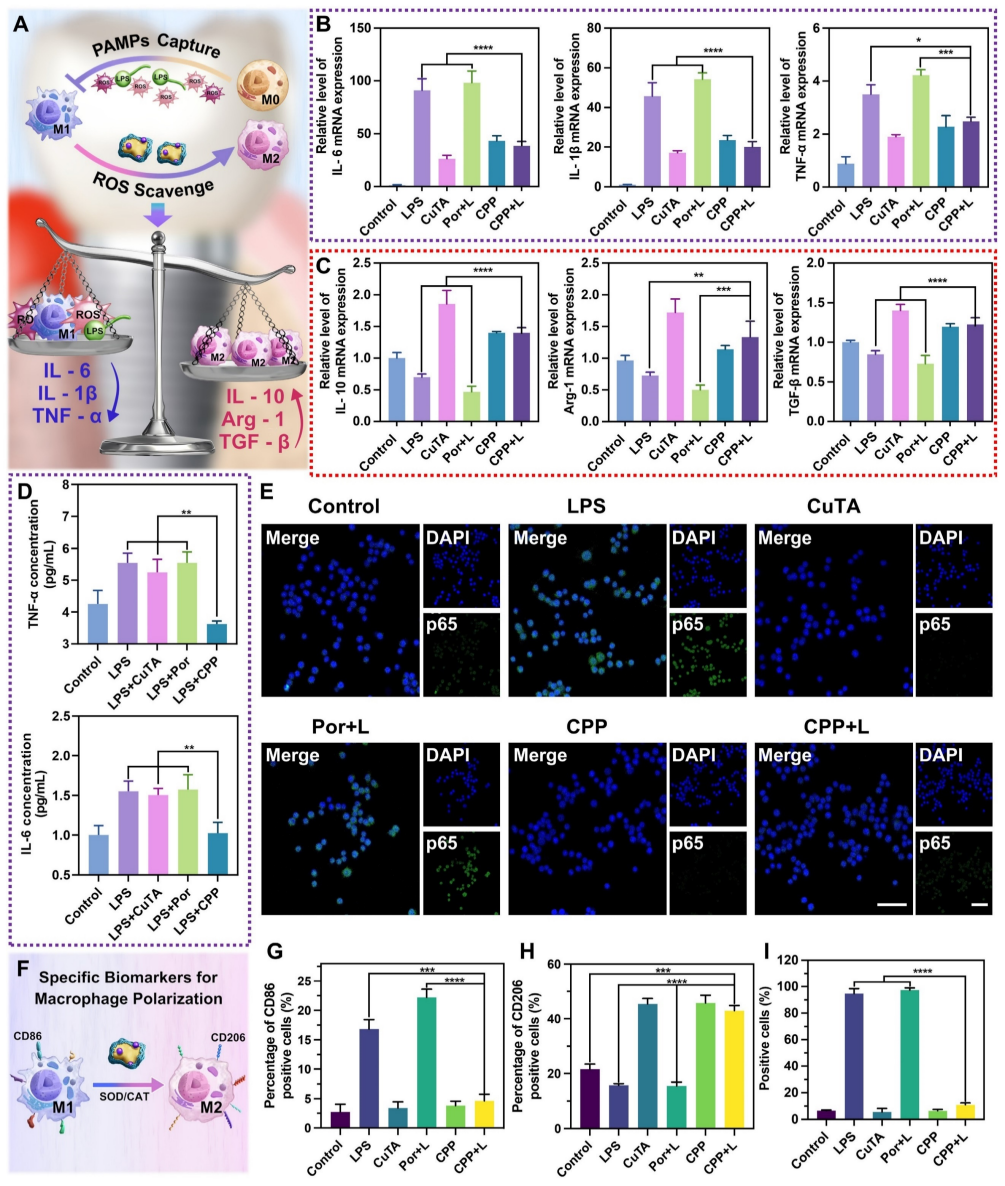
(A) Scheme of anti-inflammatory activity of CPP NPs. (B) RT-qPCR analysis of the relative mRNA level of pro-inflammatory factor (M1 markers: IL-6, IL-1β, TNF-α) and (C) anti-inflammatory factor (M2 markers: IL-10, Arg-1, and TGF-β) after different treatments. (D) The concentration of IL-6 and TNF-α in the supernatant after different treatments for 3 h. (E) Immunofluorescent images of NF-κB/p65 translocation in macrophages (scale bar: 50 μm) and (I) the quantification of positive cells. (F) Scheme of macrophage polarization regulation mediated by CPP NPs. (G) The quantification of CD86-positive cells and (H) CD206-positive cells by flow cytometry under different experimental treatments. Data are shown as mean ± SD (n = 3) (*P < 0.05, **P < 0.01, ***P < 0.001, and ****P < 0.0001).

The two most prominent functions of CPP NPs in terms of inhibiting M1 macrophage polarization are scavenging ROS through SOD/CAT enzyme activity and blocking PAMPs (Figure 6A). Through the scavenging of ROS, CPP NPs are capable of effectively alleviating the oxidative stress within macrophages. This process is crucial for regulating the inflammatory environment and counteract the excessive inflammatory responses induced by M1 macrophage polarization.

First, we stimulated RAW 264.7 cells with *P. gingivalis*-LPS for 3 h to simulate acute inflammation *in vitro*, and then added different NPs into the cells for treatment. The groups of CuTA, CPP and LPS without irradiation were employed as a comparison for their anti-inflammatory properties against PI. The groups of Por and CPP upon 660 nm laser irradiation were utilized to comparatively investigate the protection capabilities against aPDT-aggravated inflammation during the treatment. As shown in Figure 6B, LPS significantly stimulated the secretion of M1-related inflammatory cytokines such as interleukin-6 (IL-6), interleukin-1β (IL-1β) and tumor necrosis factor-α (TNF-α). In the Por+L group, the levels of these related inflammatory factors were further increased, which indicated that aPDT further aggravate the inflammatory response induced by LPS. This increase in pro-inflammatory factors has the potential to cause damage to surrounding tissues, ultimately accelerating the destruction of PI. Compared with the LPS group, the expression of M1-related inflammatory factors in the CuTA group and the CPP group was down-regulated. This indicates that CPP inherits the SOD and CAT mimic activities of CuTA and achieves the inhibition of pro-inflammatory factors by scavenging ROS generated by endogenous inflammatory stimulation. In addition, there was no significant difference in M1-related factors between the CPP+L group and the CPP group. This confirmed that the SOD and CAT enzyme activities of CPP NPs could not only scavenge ROS generated by endogenous inflammatory stimuli but also overcome the side effects of ROS generated by aPDT. This characteristic organically combined the antibacterial process mediated by aPDT and the anti-inflammatory process mediated by SOD/CAT, realizing a corresponding therapy targeting the pathogenesis of PI.

Moreover, the blocking of PAMPs helps prevent the over-activation of macrophages by external pathogens, thereby maintaining the balance of the immune response. PAMPs including LPS, peptidoglycans, and bacterial nucleic acids, can be recognized by host macrophages and activate the M1 polarization of macrophages.

In previous research, we discovered that CPP NPs had a strong electrostatic interaction with the bacterial cell membrane. PAMPs typically also exhibit negative charges. Hence, we hypothesized that CPP NPs might adsorb PAMPs through interaction with them, thereby alleviating the inflammatory response induced by PAMPs. Taking LPS as the representative of PAMPs, we first mixed CuTA, Por, and CPP with LPS respectively. After mixing, they were co-incubated with RAW 264.7 cells for 3 h to investigate the regulatory effect of CPP NPs on the inflammatory response induced by PAMPs. The results (Figure 6D) indicated that the SOD/CAT enzyme activity of CuTA had no significant effect during the process of PAMPs-induced inflammatory response. In contrast, the CPP group could significantly down-regulate the expression of TNF-α and IL-6. This suggests that CPP NPs can weaken the activation and polarization of macrophages to the pro-inflammatory M1 phenotype by effectively isolating PAMPs, further reducing the inflammatory response.

To further verify the anti-inflammatory and aPDT-protective mechanism of CPP NPs, the NF-κB signal pathway was assessed through the translocation of the NF-κB/p65 subunit. LPS can activate the NF-κB signal pathway, resulting in the high expression of inflammatory factors such as TNF-α, IL-1β, and IL-6. The translocation of the NF-κB/p65 subunit from the cytosol to the nucleus is a crucial process in the activation of the NF-κB signal pathway, and inhibiting the nuclear translocation of activated NF-κB is of great importance 55. The quantification of the positive cell ratio for each group was presented in Figure 6E and Figure 6I. The aPDT group (Por+L) had a slightly higher fluorescent expression of p65 in the nucleus (97.4%) compared to the LPS group (94.7%). However, the positive cell ratios in both the CPP and CPP+L groups were 6.46% and 10.93%, respectively, which were significantly lower than that of the LPS and Por+L group. Thus, unlike traditional aPDT treatment, our innovative aPDT strategy using CPP NPs can suppress NF-κB activation by inhibiting the translocation of the NF-κB/p65 subunit and exert a remarkable anti-inflammatory effect.

#### Promoting the transformation from M1 phenotype macrophages to M2 phenotype

In this study, factors related to M2-phenotype macrophages, such as interleukin 10 (IL-10), arginase 1 (Arg-1) and transforming growth factor beta (TGF-β) were also tested to explore whether the environmental condition with CPP addition could shift macrophage polarization from M1 to M2 phenotype in the immunoregulatory process.

Regarding M2 phenotype-associated cytokines, Figure 6C showed the mRNA expression of IL-10, Arg-1 and TGF-β in groups with different treatments. All groups containing CuTA demonstrate the characteristics of up-regulating M2-marker expression and promoting the shift of M1 phenotype to the anti-inflammatory and wound healing M2 phenotype. Macrophage polarization depends on various environmental stimuli. A deficiency in ROS production and sufficiency in SOD levels induce polarization toward the M2 phenotype, accompanied by a reduction in M1 markers and an increase in M2 markers. In cell auto-regulation, mitochondria transform from ROS production to SOD expression under environmental stimulation to dispel oxidative damage and initiate the repair function 53. During this process, M1 macrophages convert to M2 macrophages (Figure 6F). However, without external intervention, the positive transformation of cellular self-regulation is limited. CuTA, as an excellent antioxidant material, can facilitate the transformation process of macrophages through ROS scavenging and SOD mimicking. Therefore, groups containing CuTA exhibited a high level of M2-related factors. The results of flow cytometry were consistent with this trend, further verifying this point (Figure 6G-H, Figure S10A-B).

In general, CPP NPs reshape the immune microenvironment of PI through SOD/CAT-mediated ROS scavenging, blocking of PAMPs, and inhibition of the NF-κB pathway, showing remarkable anti-inflammatory activity against LPS and aPDT-induced inflammation.

### *In vivo* anti-inflammatory and antibacterial performance of CPP NPs

In this section, the biosafety of CPP NPs *in vivo* was first evaluated. According to the experimental design, CPP NPs are administered via injection into the periodontal pocket surrounding the implant. Consequently, the primary route of CPP NPs entry into the body is oral ingestion, with a minor possibility of hematogenous entry. To simulate these scenarios, we daily administered CPP NPs to rats either by gavage or tail vein injection. After a continuous one-week treatment, blood samples and major organs were collected for blood routine analyses, liver and kidney function tests (Table S3-S4), and H&E staining (Figure S11). Notably, the dosage of CPP NPs introduced into the rats through gavage or caudal vein injection in this experiment was substantially higher than that during conventional treatment. Nevertheless, neither the blood tests nor the H&E staining revealed any significant abnormalities or structural alterations, thereby validating the exceptional biological safety of CPP NPs.

**Figure 7:**
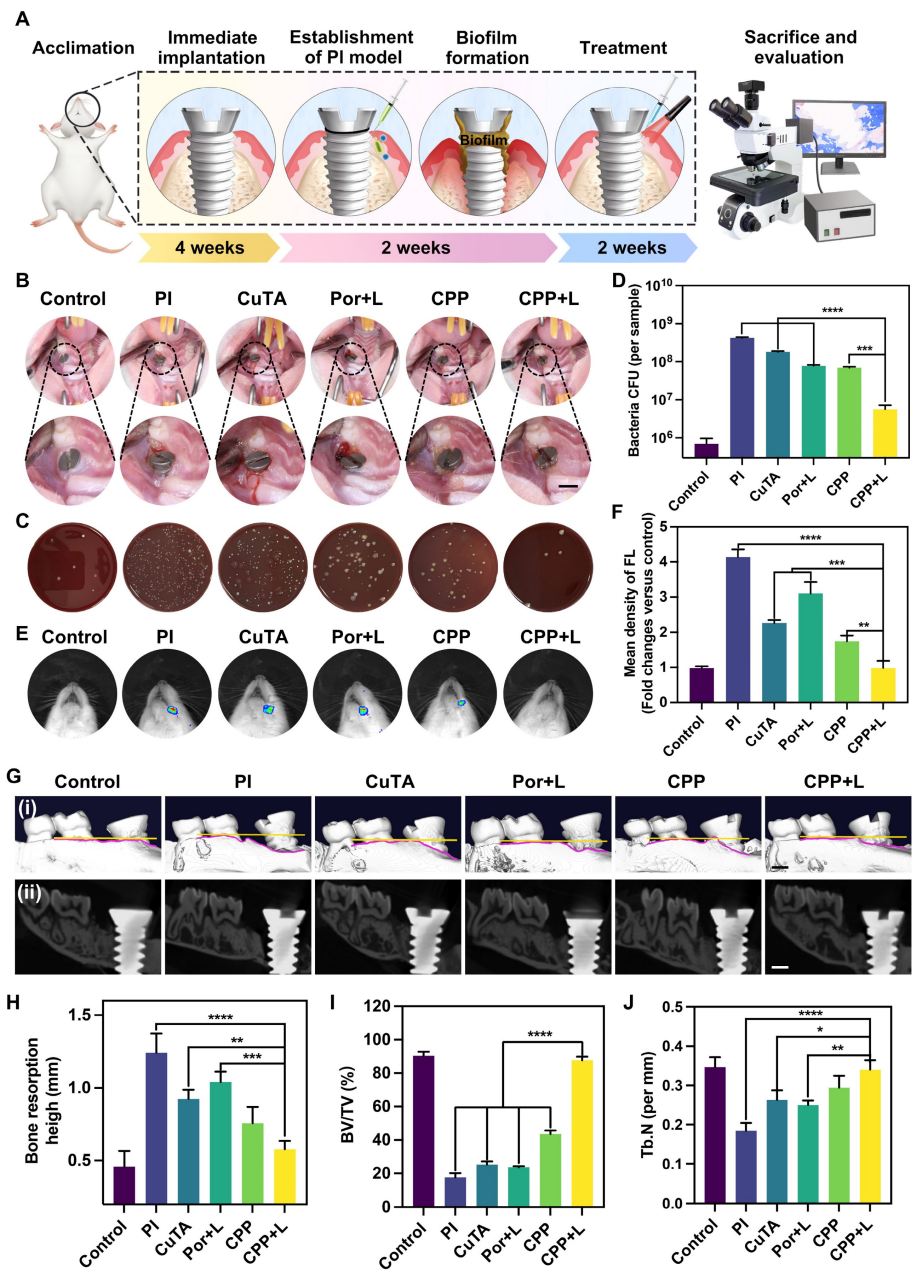
(A) Scheme diagram of the establishment of PI model and drug administration. (B) The intraoral photos for modeling rats after treatments by different NPs. Scale bar: 2 mm. (C) Represent images of colonies isolated from peri-implant tissue after different treatments, and (D) relevant quantitative analysis. (E) *In vivo* fluorescence imaging of ROS level at the site of implants with different treatments, and (F) the corresponding quantification. (G) (i) 3D reconstructed and (ii) the buccopalatal section images of the implants with different treatments by Micro-CT (scale bar: 1 mm). (H-J) The quantitative statistics of different parameters of the alveolar bone. Data are shown as mean ± SD (n = 3) (*P < 0.05, **P < 0.01, ***P < 0.001, and ****P < 0.0001).

Then, the anti-inflammatory and antibacterial activities of CPP NPs *in vivo* were investigated through the construction of PI rat model (Figure 7A). Briefly, the rats were subjected to general anesthesia using pentobarbital sodium, and local infiltration anesthesia was performed on the left maxillary first molar with lidocaine. Subsequently, the left maxillary first molar was thoroughly extracted. Next, the cavity was prepared in a step-by-step manner, followed by the implantation of the implant. 4 weeks after the implantation, PI was induced by ligating the neck of the implant with silk thread and inoculating *P. gingivalis* for 2 weeks. After the establishment of the PI rat model, evident swelling emerged in the gingiva surrounding the implant, accompanied by a dark-red coloration, food residue accumulation, and significant bleeding upon probing.

After two-week treatments, we initially captured intra-oral photographs of the rats (Figure 7B). The photographs demonstrated that, in contrast to the CPP+L group, the PI group exhibited evident gingival recession and implant threads exposure. Moreover, the gingival tissue in the PI group became soft and congested, which verified that CPP NPs effectively alleviated the clinical symptoms of PI. Meanwhile, the plaque around the implant was collected for CFU counting (Figure 7C-D). The results demonstrated that the CPP+L group exhibited the greatest reduction in CFU counts, which was over 75-fold lower than that of the PI control group. Additionally, the number of *P. gingivalis* was significantly decreased, suggesting that CPP NPs effectively eliminated the peri-implant biofilm and reduced the number of pathogenic bacteria.

Subsequently, the left maxillary bones of the rats were collected for Micro-CT imaging, CT image analysis, and three-dimensional reconstruction (Figure 7Gi-ii, Figure S12). The results indicated that, compared with the PI group, the treatments with CuTA, Por, CPP and CPP+L groups all mitigated bone resorption of PI to different extents. The therapeutic efficacy of the CPP+L group in suppressing alveolar bone resorption was 1.8 times as high as that of the Por+L group (Figure 7H), suggesting that CPP NPs achieved a more superior treatment outcome compared with the simple aPDT. Meanwhile, the bone volume fraction (BV/TV) and trabecular number (Tb.N) of LPS group decreased significantly (Figure 7I-J). And after the treatment with CPP NPs, the values of BV/TV and Tb.N were the higher compared with the other groups, indicating that CPP NPs better inhibit the destruction of the alveolar bone structure.

In addition, to explore the level of ROS around the implant after the completion of treatment, *in vivo* live animal imaging was performed using DCFH-DA as a probe (Figure 7E-F). After treatment with CPP NPs, the ROS level in the peri-implant tissues was comparable to that of the control group, confirming that CPP NPs can effectively relieve the oxidative stress state of the tissues. According to the results of CFU counting, the antibacterial efficacies of the Por+L group and the CPP group were comparable. However, the ROS level in the Por+L group was significantly increased, indicating that although aPDT eliminated a portion of bacteria, the ROS it generated was a double-edged sword and brought side effects to the tissues.

**Figure 8:**
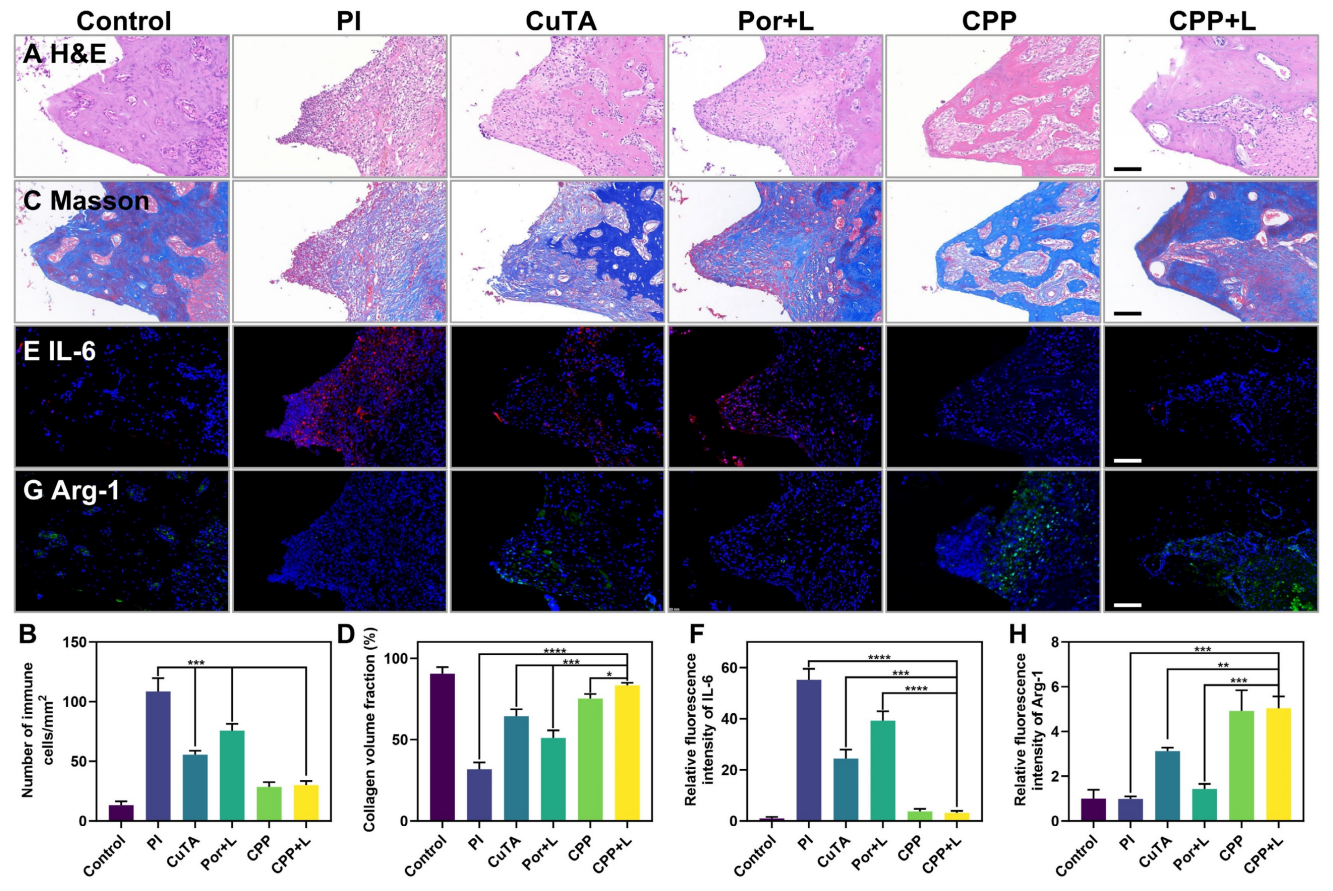
Histological level to evaluate the effect of PI treatment. Scale bar: 100 μm. (A) H&E staining images and (B) statistics of the number of immune cells. (C) Masson's staining images and (D) percentage of collagen volume fraction. (E) Immunofluorescence images (red: IL-6-positive cells, blue: nuclei) and (F) the relative immunofluorescence intensity of IL-6. (G) Immunofluorescence images (green: Arg-1-positive cells, blue: nuclei) and the (H) relative immunofluorescence intensity of Arg-1. Data are shown as mean ± SD (n = 3) (*P < 0.05, **P < 0.01, ***P < 0.001, and ****P < 0.0001).

H&E staining was used for the histological evaluation of gingival tissues to analyze the density of inflammatory cells. As shown in Figure 8A, in the inflammatory control group (PI group), infiltrated inflammatory cells such as neutrophils and macrophages were clustered. Based on the statistical analysis in Figure 8B, the CPP and CPP+L group had the least number of inflammatory cells among the experimental groups, suggesting its relatively strong inhibitory effect on local inflammation *in vivo*. Next, we examined collagen degradation at the inflammatory site by Masson staining (Figure 8C-D). It has been documented in the literature that collagenase secreted by pathogens has the capacity to disrupt collagen fibers within the tissues surrounding the implant 56. In the control group, dense and well-organized blue collagen fibrils were identified, suggesting a healthy tissue state. In comparison, most collagen fibrils in the inflammatory control group were in a degraded state, as manifested by a red hue. Notably, the collagen status in the CPP+L group was comparable to that of the control group, implying that this treatment was effective in preserving collagen integrity. This preservation is presumably due to the combined functional synergy of CPP NPs, which promotes a favorable environment for the recovery of the tissues in the vicinity of the implant.

Notably, the CPP+L group had the most obvious increase in Arg-1 and the most significant decrease in IL-6 expression (Figure 8E-H). Arg-1 is crucial for tissue repair and anti-inflammation, while IL-6 is a key pro-inflammatory cytokine. The differential expression pattern of these key inflammatory mediators clearly highlights the regulatory ability of CPP NPs for the inflammatory responses in peri-implant tissues, verifing the great potential of CPP NPs to restructure the inflammatory microenvironment* in vivo*.

## Conclusion

To overcome the limitations of aPDT in the treatment of PI, CPP NPs with both antibacterial and anti-inflammatory functionalities were engineered with a focus on the spatiotemporal modulation of ROS. The remarkable therapeutic outcomes of CPP NPs can be summarized in two pivotal aspects. On the one hand, the highly positively charged surface of CPP NPs markedly enhances the penetration and distribution within the biofilm as well as preferential selectivity for bacteria, thereby leading to the eradication of the biofilm via aPDT-mediated ROS burst. On the other hand, CPP NPs can scavenge ROS in PI tissue and residual ROS post-aPDT to suppress the inflammatory responses, regulate macrophage polarization, and facilitate tissue regeneration. This meticulously devised strategy holds the promise of proffering a new approach for augmenting the treatment efficacy of PI in future clinical applications.

## Materials and Characterizations

### Materials

Copper sulfate pentahydrate, tannic acid (TA), sodium hydroxide, ethyl alcohol, glutaraldehyde, 3-aminopropyltriethoxysilane (APTES), protoporphyrin (Por), N-Hydroxysuccinimide (NHS), ethylenediamine (EDC), ε-polylysine (ε-PL), riboflavin, methionine, nitro blue tetrazolium (NBT), 1,3-diphenylisobenzofuran (DPBF), hydrogen peroxide (H₂O₂), crystal violet, Nile-Red, and ferrous sulfate (FeSO₄) were obtained from Aladdin Biochemical Technology Co., Ltd. Cu^2+^ Colorimetric Assay Kit was obtained from Elabscience. 2',7'-dichlorofluorescein diacetate (DCFH-DA), N, N-dimethylaniline N-oxide (DMAO), anti-NF-κB p65 primary antibodies, and Alexa 488-conjugated IgG secondary antibodies were purchased from Beyotime. Sulfo-Cyanine5.5 N-hydroxysuccinimide ester (Cy5.5-NHS), and Hoechst 33342 were purchased from Meilun Bio. PE-conjugated anti-mouse CD86 and APC-conjugated anti-mouse CD206 were obtained from BioLegend.

### Characterizations

The hydrated particle size distribution, PDI, zeta potential, and morphology were determined by dynamic light scattering (DLS, Nano ZS90, UK) and transmission electron microscopy (TEM, JEM2010, Japan). The UV-vis spectra of the intermediate and final products were measured using a UV-vis-NIR spectrometer (PerkinElmer LAMBDA 950, from the USA). The crystal structures of the samples were confirmed by XRD (Bruker D8 Advance, Germany) and XPS (Thermo Fisher Scientific, United States). CCK-8 and Elisa assays were carried out with a microplate reader (Sunrise, USA). Fourier transform infrared spectra were obtained via a Fourier transform infrared spectrometer (FTIR, Nicolet, Nicolet 5700, US). The live/dead staining of biofilms, intracellular ROS generation, and immunofluorescence experiments were visualized under a confocal laser scanning microscope (CLSM, Leica, Germany).

## Methods

### Synthesis of CuTA-Por@ε-PL (CPP)

#### Synthesis of CuTA

CuTA nanosheets were fabricated through the coordination of copper ions with polyphenol group-containing TA, following a previously reported synthesis method 32. Specifically, 54 mg of TA and 1750 mg of copper sulfate pentahydrate were dissolved in 100 mL of deionized water. The pH of the mixture was adjusted to 7.4 by adding approximately 5 mL of 2 M sodium hydroxide solution. Subsequently, the solution was heated to 60 °C in an oil bath and stirred for 5 h. The product was obtained by centrifugation at 5000 rpm for 5 min and then washed three times with deionized water and once with absolute ethyl alcohol. After drying under vacuum in an oven at 60 °C for 2 h, the product was stored at 4 °C for subsequent experiments.

#### Synthesis and* in vitro* ROS generation of CuTA-Por

Amino-functionalized nanosheet was produced in the presence of APTES. 100 mg of CuTA was distributed in 50 mL ethyl alcohol, and 200 μL of APTES was added to the nanoparticle suspension. The solution was heated to 60 °C in an oil bath and stirred for 4 h. The product, “CuTA-NH_2_”, was obtained by centrifugation at 5000 rpm for five min and then washed three times with absolute ethyl alcohol, and dried under vacuum in an oven at 60 °C for 2 h.

CuTA-Por was prepared through amidation reaction. Por (5 mg, 0.007 mmol), NHS (4.1 mg, 0.036 mmol), and EDC (13.6 mg, 0.071 mmol) were dissolved in 30 mL of deionized water. Under a nitrogen atmosphere, the mixture was stirred at 25 °C for 30 min to activate the carboxyl group of Por. Subsequently, 20 mL CuTA solution (containing 100 mg of CuTA) was added to the activated Por solution and stirred for 6 h at 25 °C under nitrogen protection. It could be observed that the reaction solution gradually changed from green to purple. After the reaction, the solution was centrifuged (5000 rpm, 5 min), washed three times with deionized water. After drying, the product CuTA-Por (Por/CuTA mass ratios 1:20) was obtained. Similarly, CuTA-Por with different Por/CuTA mass ratios (1:10, 1:40, 1:80) was fabricated.

The generation of extracellular ROS was evaluated by using 1,3-diphenylisobenzofuran (DPBF) as the singlet oxygen sensors. In a typical experiment 57, DPBF was added to CuTA-Por solutions with different Por/CuTA mass ratios (1:10-1:80) to record the absorbance intensity at 420 nm. The absorbance was measured every 30 seconds during 660 nm laser irradiation.

#### Preparation of CPP

CPP NPs were formed through electrostatic interaction. In brief, an aqueous solution of ε-PL (25 mL, 20 mg/mL) and an aqueous solution of CuTA-Por (25 mL, 2 mg/mL) were mixed. Subsequently, the mixture was sonicated for 30 min and stirred at 25 °C for 6 h. After that, the product was purified by several cycles of washing and centrifugation using deionized water and dried by lyophilization. CPP with different mass ratios of ε-PL/CuTA-Por (1:5 and 1:20) was fabricated in a similar manner.

### Stability, dispersion and the release of Cu^2+^of CPP NPs

The stability of the CPP was evaluated by incubating it in PBS, DMEM (containing 10% serum) and artificial saliva at 37 °C for 7 days. The size distribution and PDI was measured using DLS.

The release of Cu²⁺ was determined using a Cu²⁺ Colorimetric Assay Kit. First, a standard curve for Cu²⁺ was prepared. 200 mg of CPP was dissolved in 5 ml of PBS (pH 6.0, 7.0, 7.4), and then transferred into a dialysis bag (500 MWCO). Silk thread was used for ligation to ensure no liquid leakage. Subsequently, the dialysis bag was placed in 45 ml of external PBS solution with pH values of 6.0, 7.0, and 7.4 respectively, and the setup was placed on a constant temperature shaker at 37 °C. Samples were taken at different time points to detect the copper ion concentration in the external solution. After sampling, fresh PBS was added to maintain the volume of the external solution at 45 mL.

### Evaluation of the biosafety of CPP NPs

The biosafety of CPP was first evaluated by CCK-8 assay and hemolysis assay. HGFs cells and L929 cells were first seeded in 96-well plates and allowed to culture overnight. Subsequently, CPP NPs of varying concentrations were added and left for 24 h. Once the treatment was completed, the old medium was replaced with fresh medium containing CCK-8, and the cells were incubated for another 3 h. Eventually, the cell viability was determined by a microplate reader at a wavelength of 450 nm. For hemolysis assay, red blood cells were separated via centrifugation at 1500 rpm for 15 min. A red blood cell suspension was then generated by rinsing the red blood cells with PBS three times and diluting them tenfold. The positive control (+) was formulated using distilled water, while the negative control (-) was made with PBS. An equal volume of the red blood cell suspension was combined with the sample solutions of diverse concentrations to be evaluated, incubated at 37°C for 2 h, and then centrifuged at 1500 rpm for 15 min. Subsequently, the supernatant was transferred to a 96-well plate, and the absorbance was determined at 576 nm with a microplate analyzer.

The biocompatibility of CPP *in vivo* was further evaluated. A total of nine 6-week-old male rats were randomly assigned to three groups. To mimic the potential pathways by which CPP could enter the body during the administration process, aside from the control group, one group received daily oral gavage of the CPP solution, while the other group was subjected to daily tail vein injection of the CPP solution. Following one week treatment, the rats were humanely euthanized. Subsequently, blood samples were collected for blood routine examination and assessments of liver and kidney function. Moreover, the major organs (heart, liver, spleen, lungs, and kidneys) were harvested for H&E staining to examine potential histological changes.

### ROS leverage based on CPP NPs

#### *In vitro* ROS generation of CPP

Similar to the method in 2.1.2, the extracellular ROS generation of CPP was evaluated by using DPBF. DPBF was added to Por, CuTA-Por, and CPP solutions to record the absorbance intensity at 420 nm. The absorbance was measured per minute during 660 nm laser irradiation.

#### *In vitro* ROS scavenging of CPP

##### Scavenging ^•^O₂⁻ *in vitro*

The **^•^**O₂⁻ scavenging capacity of CPP NPs was assessed by determining the inhibition ratios of the photoreduction of NBT 32. Here, 20 μM riboflavin, 12.5 mM methionine, 75 μM NBT, and CPP NPs with different concentrations were dissolved in PBS (pH 7.4). Then, the mixtures were exposed to ultraviolet radiation for 15 min. Following illumination, **^•^**O₂⁻ reduced NBT to a blue product that had an absorption peak at 560 nm. This product was further quantified through UV-vis absorption spectroscopy. Samples containing riboflavin, methionine, and NBT in the absence and presence of ultraviolet illumination were designated as the negative control and positive control respectively. All experiments were carried out in the dark. The **^•^**O₂⁻ scavenging capacity of CuTA, and CPP NPs following 5 min 660 nm laser irradiation were detected via the same method.

##### Scavenging ^•^OH *in vitro*

The **^•^**OH scavenging efficiency of CPP NPs was evaluated by monitoring the quantity of **^•^**OH in salicylic acid (SA). Firstly, 2 × 10⁻⁴ M hydrogen peroxide (H₂O₂) and 2 × 10⁻⁴ M ferrous sulfate (FeSO₄) were combined to generate **^•^**OH through a Fenton reaction. Subsequently, CPP NPs at different concentrations were added to the solution to eliminate **^•^**OH. Finally, the remaining **^•^**OH was detected by adding 1 × 10⁻³ M SA. The **^•^**OH oxidized SA to 2,3-dihydroxybenzoic acid with a purple color. The absorption peak at 510 nm was further determined by UV-vis absorption spectroscopy. The **^•^**OH scavenging efficiency of CuTA, and CPP NPs following 5 min 660 nm laser irradiation were detected in the same way.

##### Catalase-like activity of CPP NPs

The catalase-like activity of CPP NPs was investigated by the rate of H₂O₂ clearance. In brief, H₂O₂ and CPP NPs of different concentrations were mixed into 5 mL of PBS (pH 7.4). The generated oxygen (O₂) was quantitatively measured with a dissolved oxygen meter. The catalase-like activity of CuTA, and CPP NPs following 5 min 660 nm laser irradiation were also investigated.

##### Dynamic monitoring of the generation and scavenging of ROS *in vitro*

As per the literature 24, 2',7'-dichlorofluorescein diacetate (DCFH-DA) was utilized to measure the levels of ROS inside and outside cells. The experimental group was categorized into five groups: the control group (Control), the CuTA group (CuTA), the CPP group (CPP), the Por with 5 min 660 nm laser irradiation group (Por+L), and the CPP with 5 min 660 laser irradiation group (CPP+L). The stock solutions for each of these five groups were prepared separately. The Por+L group and the CPP+L group were subjected to 660 nm laser irradiation for 5 min, whereas the other groups remained untreated. Subsequently, 180 μL of solution was promptly taken from the stock solution and added to the well plate, followed by the addition of DCFH-DA. The fluorescence imaging was immediately recorded on the IVIS^®^ Imaging System (with an excitation wavelength of λex = 488 nm and an emission wavelength of λem = 525 nm), and this was marked as “0 min”. Subsequently, every 3 min, 180 μL of solution was taken from the stock solutions and added to the well plate, DCFH-DA was added, and the aforementioned detection process was repeated.

The detection of intracellular ROS levels was also divided into the above five groups: Control, CuTA, CPP, Por+L, CPP+L. Human gingival fibroblasts (HGFs) were seeded in confocal microscopy dishes (35 mm) at a density of 2×10⁵ cells/dish and cultured at 37°C for 24 h. Then, PBS, CuTA, Por, and CPP were added respectively, with or without laser treatment. After irradiation, cells were incubated for 6 h at 37 °C. Subsequently, DCFH-DA was added and incubated for 20 min. Then, cells were fixed with 4% paraformaldehyde for 10 min. Finally, the cells were stained by DAPI for 5 min and visualized by confocal laser scanning microscopy (CLSM).

### Evaluation of the biofilm penetration and bacterial selectivity mediated by CPP NPs

#### Biofilm penetration and dispersion mediated by CPP NPs

For the formation of mature *P. gingivalis* biofilms, the microbial concentration was set to 10^8^ CFU/mL in confocal microscopy dishes and then cultured in an anaerobic medium at 37°C for 96 h 46. To evaluate the penetration ability of NPs into biofilms, CuTA were labeled with Cy5.5 to obtain CuTA-Cy5.5 and then synthesized CuTA-Cy5.5@ε-PL (CCP). CuTA-Cy5.5 and CCP were used to represent CuTA-Por and CPP NPs respectively. Then, 1 milliliter of CuTA-Cy5.5 and CCP solutions were added to the biofilms and incubated for 0.5, 1, and 2 h respectively. Upon completion of incubation, the biofilms were washed three times with PBS to eliminate NPs that were not bound to the biofilms. Subsequently, the biofilms were stained with DMAO in the dark for 20 min. The penetration of NPs into the biofilms was observed using CLSM.

#### Selective absorption of CPP NPs on cells and bacteria

The HGFs cells were stained with Hoechst 33342 for 20 min and then washed with PBS, the *P. gingivalis* were stained with Nile-Red for 20 min and washed with PBS as well 33. The HGFs cells and *P. gingivalis* were combined in PBS at concentrations of 10^5^ cells/mL and 10^7^ CFU/mL, respectively. Subsequently, CCP were added to the co-incubation solution and incubated for another 30 min. Finally, the samples were quantified by flow cytometry.

HGFs at a concentration of 10^5^ cells/mL and *P. gingivalis* at a concentration of 10^7^ CFU/mL were individually co-incubated with CPP NPs of different concentrations for 30 min. After that, they were centrifuged separately at 5000 revolutions for 3 min and washed three times to remove unbound CPP NPs. The cells/bacteria were collected, and after resuspension, the impact of CPP NPs on the surface charge of cells/bacteria was explored by measuring their Zeta potential.

In addition, after 30 min co-incubating of *P. gingivalis* with CPP NPs, the bacteria were fixed with glutaraldehyde solution for 30 min and then observed by TEM.

### Evaluation of antibacterial and anti-biofilm properties of CPP NPs

#### Evaluation of antibacterial ability of CPP NPs to free bacteria

Various concentrations of CPP NPs were mixed with 1 mL of *P. gingivalis* bacterial solution (10^6^ CFU/mL). Following 5 min 660 nm laser irradiation, co-incubation was carried out for 24 h. After gradient dilution, the solution was uniformly spread on blood agar plates and then cultured anaerobically at a constant temperature of 37 °C for colony counting.

For TEM imaging, the bacterial suspension was fixed with 2.5% by weight glutaraldehyde. Subsequently, images were taken to observe the bacterial morphology and the interaction with CPP NPs.

Similarly, with the concentration of CPP fixed at 300 μg/mL, five groups (control, CuTA, Por+L, CPP, and CPP+L group) with or without laser irradiation were established to further investigate the antibacterial efficacy of CPP NPs by CFU counting and TEM.

#### Nucleic acid and protein leak assay

The *P. gingivalis* bacterial solution was adjusted to a concentration of 10^8^ CFU per milliliter and treated with different NPs and laser irradiation 55. Subsequently, the bacterial suspension was collected every 10 min and the supernatant was diluted. Finally, the release of nucleic acid was determined by measuring the optical density value at 260 nanometers. The protein leakage amounts of bacteria after treatment were measured using the BCA Protein Assay Kit.

#### Evaluation of antibiofilm properties

Five groups were established: control group, CuTA group, Por+L group, CPP group, and CPP+L group. The wells containing forming or established biofilm were treated with different NPs and then irradiated with 660 nm laser (1 W/cm^2^, 5 min). The control group, CuTA group, and CPP group did not receive laser treatment.

For CFU counting, the attached biofilm was removed through ultrasonic treatment after different treatments. Subsequently, it was continuously diluted tenfold. Then, 100 microliters of the diluted bacterial solution were spread on blood agar and anaerobically cultured in a constant temperature incubator.

For live/dead fluorescent staining, the biofilm was washed to eliminate non-adherent bacteria. Subsequently, a mixture of SYTO 9 (2.5 μM) and propidium iodide (2.5 μM) was prepared and employed to stain each sample for 20 min in accordance with the manufacturer's instructions. 3D-images of the biofilm were acquired using CLSM.

For crystal violet staining, the treated biofilms were first fixed for 15 min. After being air-dried naturally, a 1 mg/mL crystal violet solution was added to stain them for 15 min. Subsequently, the excess dye was thoroughly washed off. Finally, the stained biofilms were dissolved with 95% ethanol, and the absorbance at 590 nm was measured using a microplate reader.

For the evaluation of gene expression of the *P. gingivalis* extracellular proteins. Specifically, genes such as FimA II, FimA IV, RgpA, RgpB, and Kgp were analyzed using the RT-qPCR technique. The primers were synthesized by BGI TECH SOLUTIONS (BEIJING LIUHE) Co., Ltd, and the details are provided in Table S1. The relative gene expression was determined by the 2^-ΔΔCt^ method. For data normalization, the 16 S rRNA gene was employed as an internal control, with the Ct value of the control group acting as the calibrator. All experiments were replicated three times.

For transcriptomic analysis of *P. gingivalis* after the treatment of CPP NPs, the established *P. gingivalis* biofilm were treated with CPP NPs and irradiated with 660 nm laser. After being treated, the biofilm was harvested by centrifugation (10000 rpm, 3 min), then frozen in liquid nitrogen for 15 min and put into dry ice for further RNA extraction.

### Anti-inflammatory activities of CPP NPs against LPS and aPDT-induced proinflammation

#### The phenotype switch of macrophage polarization

The M1/M2 phenotype of macrophage was examined by detecting related cytokines. *P. gingivalis*-LPS was chosen as the stimulus to create the basic inflammation status and mimic the PI condition. RAW 264.7 cells were seeded at a density of 1×10^5^ cells/well in a 6-well plate and cultured for one day. Subsequently, *P. gingivalis*-LPS (1 μg/mL) was used to stimulate the cells for 3 h to mimic acute inflammation *in vitro* before adding different NPs. The groups of CuTA, CPP and LPS without irradiation were employed as a comparison for their anti-inflammatory properties against PI. The groups of Por and CPP upon 660 nm laser irradiation were utilized to comparatively investigate the protection capabilities against aPDT-aggravated inflammation during the treatment.

After a 24-hour incubation period that followed, the total RNA of macrophages was extracted using an RNA extraction kit. The M1/M2 phenotype inflammatory response of RAW 264.7 was assessed through the mRNA expression level of chemokine interleukin-1β (IL-1β), chemokine interleukin-6 (IL-6), tumor necrosis factor-α (TNF-α), chemokine interleukin-10 (IL-10), transforming growth factor-β (TGF-β), and arginine-1 (Arg-1) by employing qPCR. The data was normalized to the housekeeping gene β-actin. The primers sequences used in the study are provided in Table S2.

The expression of macrophage surface markers was measured by flow cytometry. After RAW 264.7 cells were inoculated, cultured, and treated in the same way as previously mentioned, the cells were collected and stained with PE-conjugated anti-mouse CD86 and APC-conjugated anti-mouse CD206 for 15 min at room temperature in the dark. The cells were washed three times with staining buffer (PBS/2% FBS) and then resuspended before being detected by the instrument.

#### The blocking inflammatory response induced by pathogen-associated molecular patterns (PAMPs)

Using LPS as a typical PAMPs, we investigated the ability of CPP NPs to block the inflammatory response induced by PAMPs. RAW 264.7 cells were seeded at a density of 1×10^5^ cells/well in a 6-well plate and cultured for one day. Subsequently, *P. gingivalis*-LPS (1 μg/mL) was initially mixed with different NPs. Then, the mixture was added to the cells for a 3-hour stimulation. The concentrations of IL-6 and TNF-α in the supernatant of RAW 264.7 cells after treatment were detected by ELISA kits.

#### Inhibition of LPS and aPDT-induced NF-κB activation in macrophages

To further validate the anti-inflammatory and aPDT-protective mechanism of CPP NPs, the NF-κB signal pathway was assessed through the translocation of the NF-κB/p65 subunit. RAW 264.7 cells (1×10^5^ cells/well) were seeded into a 6-well plate, and cultured for 24 h. The seeded cells were stimulated with *P. gingivalis*-LPS (1 μg/mL) for 3 h, and different NPs were then added to the wells with or without laser irradiation and incubated for 24 h. After fixation with 4% paraformaldehyde for 10 min, the cells were treated with a 0.25% Triton X-100 for 10 min, and then blocked with blocking goat serum for 1 h at 37 °C. Subsequently, the cells were incubated with rabbit anti-NF-κB p65 primary antibodies (1:500) for 1.5 h at 37 °C, followed by incubation with goat anti-rabbit Alexa 488-conjugated IgG secondary antibodies (1:1000) for 1 h at 37 °C. The DAPI was utilized to stain the cell nucleus by incubating for 5 min in the dark. Finally, CLSM was used for imaging.

### *In vivo* evaluation of the antibacterial and anti-inflammatory properties of CPP NPs

#### Establishment animal model and administration

All animal experiments have obtained ethical review and approval from the Ethics Committee of the Affiliated Hospital of Qingdao University. Six-week-old male Sprague-Dawley rats were procured from Beijing HFK Bioscience Co. Ltd. Following one week of adaptive feeding, the left maxillary first molars of the rats were completely extracted, and implants were promptly inserted immediately after the extraction. Four weeks later, upon the formation of osseointegration of the implants, the rats were randomly allocated into six groups, namely Control, PI, CuTA, Por+ L, CPP, and CPP+L. With the exception of the Control group, the remaining groups were subjected to treatment involving silk thread ligation around the implant neck and inoculation of *P. gingivalis* for a period of two weeks to construct the PI model. Subsequently, the Control and PI groups remained untreated, whereas the other groups were administered with the corresponding substances once a day into the peri-implant pocket. Additionally, the Por+L and CPP +L groups were irradiated with laser for a duration of five min. After two weeks of treatment, the rats were euthanized, and the left maxillary bones were harvested for micro-CT scanning and immunohistochemical analysis.

When calculating the height of implant bone resorption (Figure S12), since the mesial and distal bone heights of the implant were flush with the crest of the alveolar ridge of the adjacent maxillary second molar in rats at the initial implantation of the implant, the crest of the alveolar ridge of the second molar was taken as the baseline (blue line). The heights of bone recession at the mesial and distal ends of the implant were measured respectively and marked as A and B, and the average value of A and B was used to represent the height of implant bone recession.

#### Live animal fluorescence imaging

The ROS level of the peri-implant tissue with different treatments was studied according to the similar methods in the literature 24. After the 2-week treatment was completed, the rats in each group were anesthetized by pentobarbital sodium. Subsequently, the ROS probe DCFH-DA was intravenously injected at a dose of 1.8 mg/kg for 30 min. After that, the fluorescence imaging was recorded with an IVIS^®^ Imaging System (excitation filter: 525 nm; emission filter: 495 nm, PerkinElmer).

### Statistical analysis

All data are presented as mean ± SD. Student's unpaired t-test and two-way ANOVA were used for statistical analyses. Significance levels were set as *P<0.05, **P<0.01, ***P<0.001, and ****P<0.0001.

## Supplementary Material

Supplementary figures and tables.

## Abbreviations

PI: peri-implantitis
aPDT: Antibacterial photodynamic therapy
PS: photosensitizers
Ce6: Chlorin e6
ROS: reactive oxygen species
EPS: extracellular polymeric substances
SOD/CAT: dismutase/catalase activities
CuTA NS: Copper tannic acid coordination nanosheet
PAMPs: pathogen-associated molecular patterns
P. gingivalis: Porphyromonas gingivalis
DPBF: 1,3-diphenylisobenzofuran
SA: salicylic acid
•OH: hydroxyl radicals
H₂O₂: hydrogen peroxide
Fim: Fimbriae
DEGs: differentially expressed genes
GO: Gene Ontology
KEGG: Kyoto Encyclopedia of Genes and Genomes
TLR4: Toll-like receptor 4
NOX2: nicotinamide adenine dinucleotide phosphate oxidase
IL-6: interleukin-6
IL-1β: interleukin-1β
TNF-α: tumor necrosis factor-α
Arg-1: arginase 1
TGF-β: transforming growth factor beta
BV/TV: bone volume fraction
Tb.N: trabecular number
TA: tannic acid
APTES: 3-aminopropyltriethoxysilane
NHS: N-Hydroxysuccinimide
EDC: ethylenediamine
NBT: nitro blue tetrazolium
FeSO₄: ferrous sulfate
DMAO: N, N-dimethylaniline N-oxide
DCFH-DA: 2',7'-dichlorofluorescein diacetate
DLS: dynamic light scattering
TEM: transmission electron microscopy
CLSM: confocal laser scanning microscope
CCP: CuTA-Cy5.5@ε-PL
