# Supplementary material for: A versatile nanoplatform with excellent biofilm permeability and spatiotemporal ROS regulation for peri-implantitis treatment
Source: Theranostics. 2025 Feb 24;15(8):3490–516. doi: 10.7150/thno.108830 (PMC11905146; doi:10.7150/thno.108830)
Supplement: Supplementary file 1 — Supplementary figures and tables. [file thnov15p3490s1.pdf]

Supporting Information

A Versatile Nanoplatform with Excellent Biofilm Permeability and Spatiotemporal ROS Regulation for Peri-Implantitis Treatment

Zeyu Han[1,2#], Ying Li[1,2#], Xin Zhan[1,2], Ming Sun[1,2], Yan Liang[3], Mujie Yuan[1,2], Yong Sun[3], Jie Cao[3], Baodong Zhao[1] and Fan Li[1,3]**

[1]Department of Oral Implantology, The Affiliated Hospital of Qingdao University, Qingdao University, Qingdao 266000, P. R. China.

[2]School of Stomatology, Qingdao University, Qingdao 266000, P. R. China

[3]Department of Pharmaceutics, School of Pharmacy, Qingdao University, Qingdao 266021, P. R. China

#These authors contributed equally in this work.

* To whom correspondence should be addressed, E-mail: zbd315@sina.com; lifan911017@qdu.edu.cn.

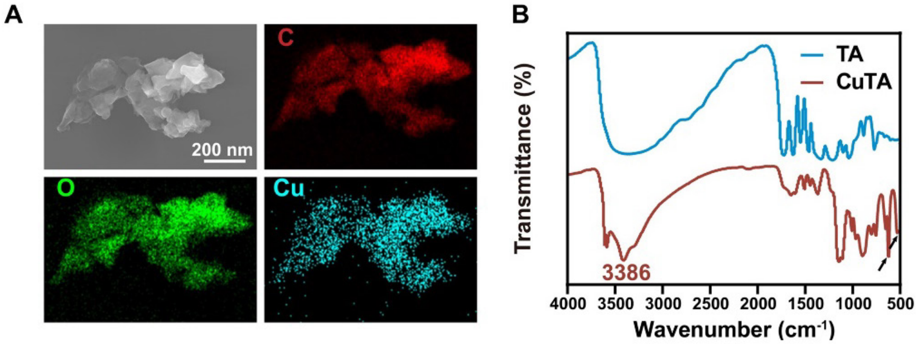

Figure S1. Characterization of CuTA. (A) SEM image and EDS-mapping analysis of CuTA. (B) FTIR of TA and CuTA.

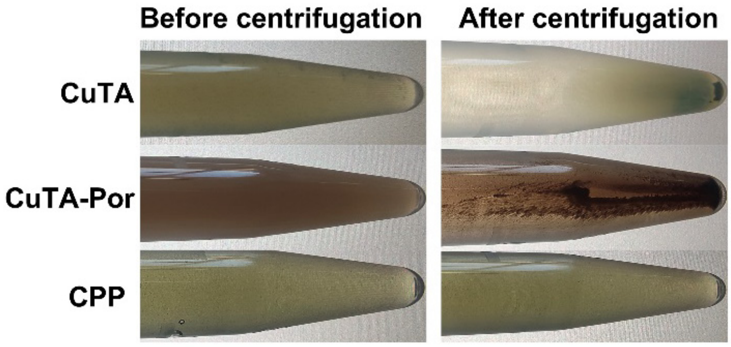

Figure S2. Images of CuTA, CuTA-Por, and CPP NPs before centrifugation and after 5 min centrifugation.

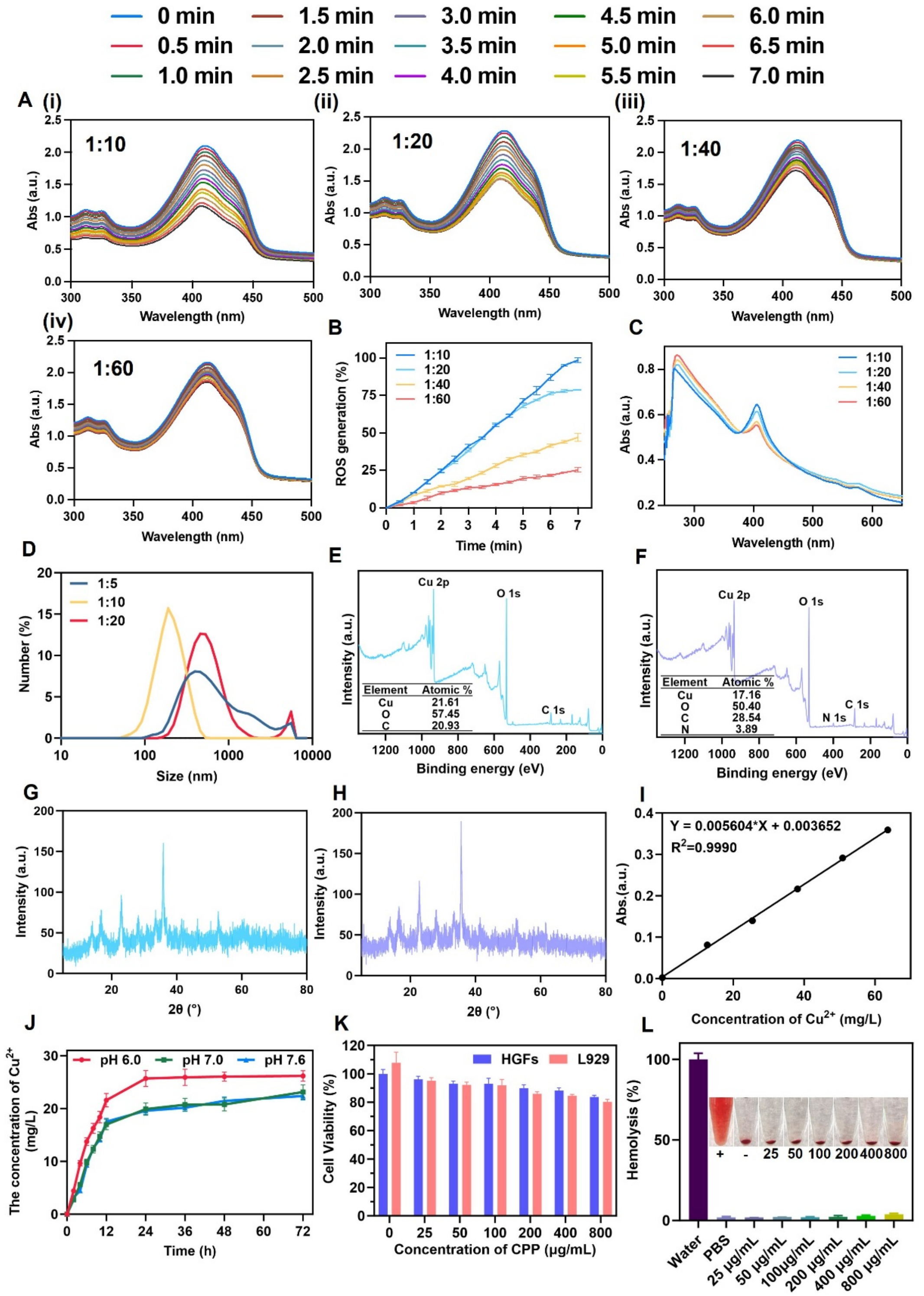

Figure S3. (A) Characterization of the *in vitro* photodynamic properties of CuTA-Por with different Por/CuTA ratios and (B) normalization analysis. (C) UV–vis spectra of CuTA-Por with different Por/CuTA ratios. (D) Hydrated particle size of CPP with different CuTA-Por/ε-PL ratios. (E) XPS spectrum of CuTA and (F) CuTA-Por. (G) XRD pattern of CuTA and (H) CuTA-Por. (I) Standard curve of Cu^{2+} and (J) the release

of Cu^{2+} from CPP at different pH. (K) CCK-8 assay of HGFs and L929 cells treated with different concentrations of CPP NPs. (L) Hemolysis assay of CPP NPs at different concentrations.

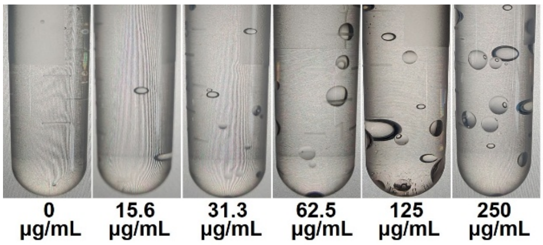

Figure S4. Images of 3% hydrogen peroxide solution after treatment with different concentrations of CPP NPs.

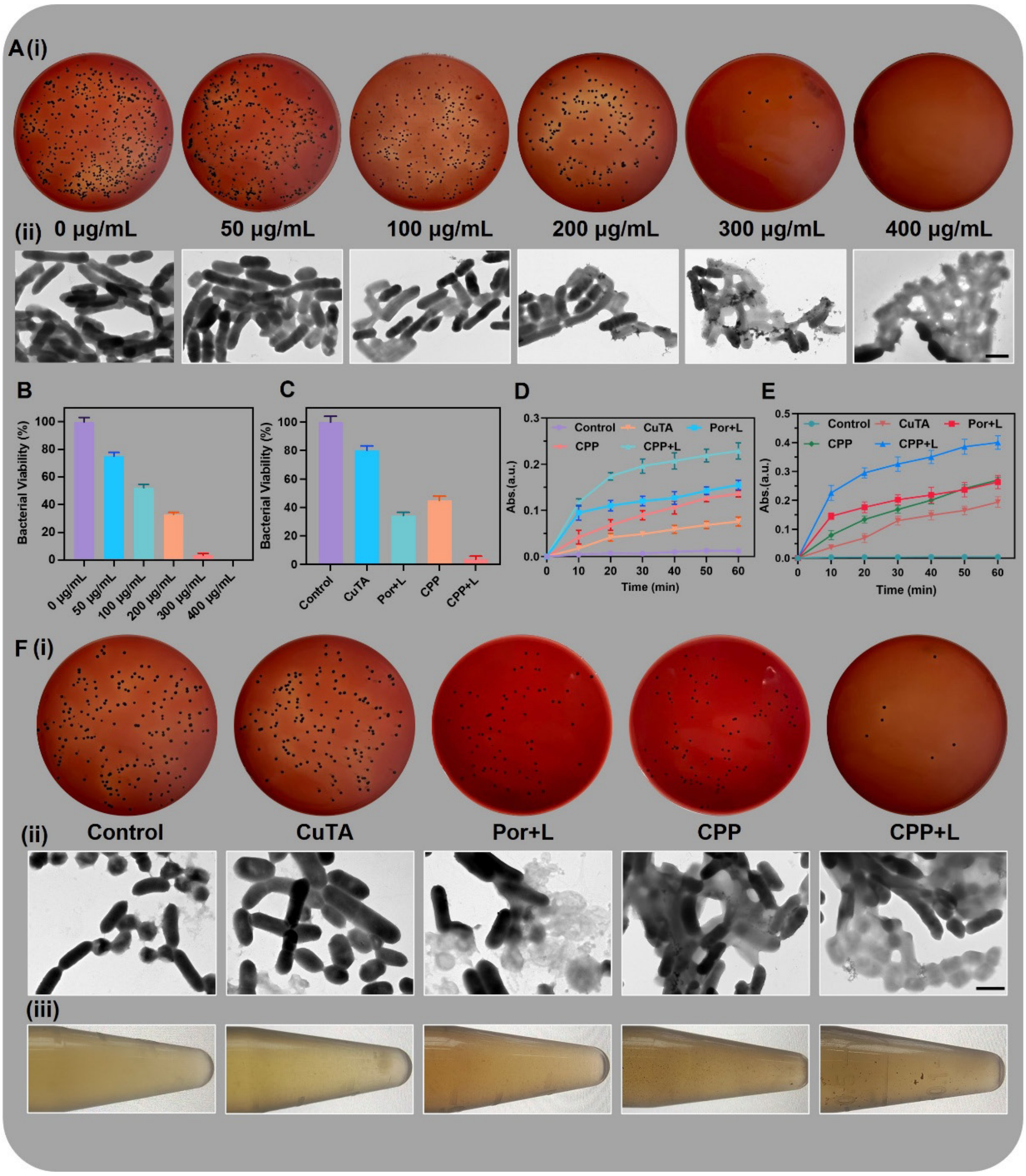

Figure S5. (A) (i) Images of colonies, (ii) TEM images, and (B) quantitative analysis of *P. gingivalis* after treatment with different concentrations of CPP NPs. Scale bar: 1 μm. (C) Quantitative analysis of *P. gingivalis* after different treatments. (D) Nucleic acid leak assay of *P. gingivalis*, the absorbance curves of *P. gingivalis* suspensions after treated with various NPs at 260 nm. (E) Protein leak assay of *P. gingivalis*. (F) (i) Images of colonies, (ii) TEM images, and (iii) photos of *P. gingivalis* after different treatments. Scale bar: 1 μm.

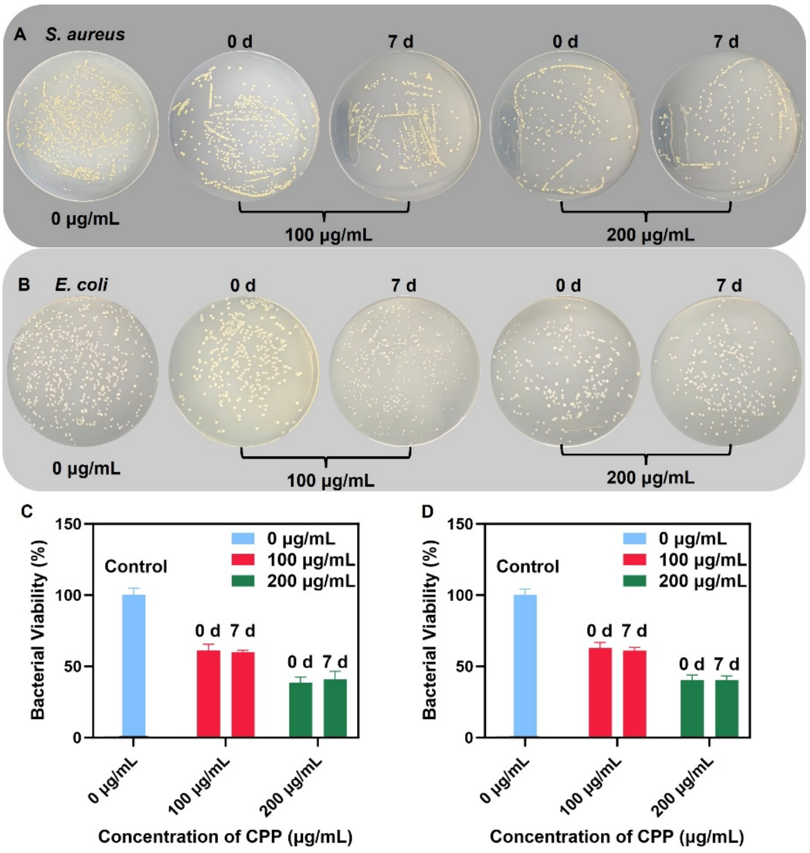

Figure S6. Evaluation of the antibacterial stability of CPP against *S. aureus* and *E. coli* after 7 days of incubation. (A) Images of *S. aureus* colonies and (C) quantitative analysis. (B) Images of *E. coli.* colonies and (D) quantitative analysis.

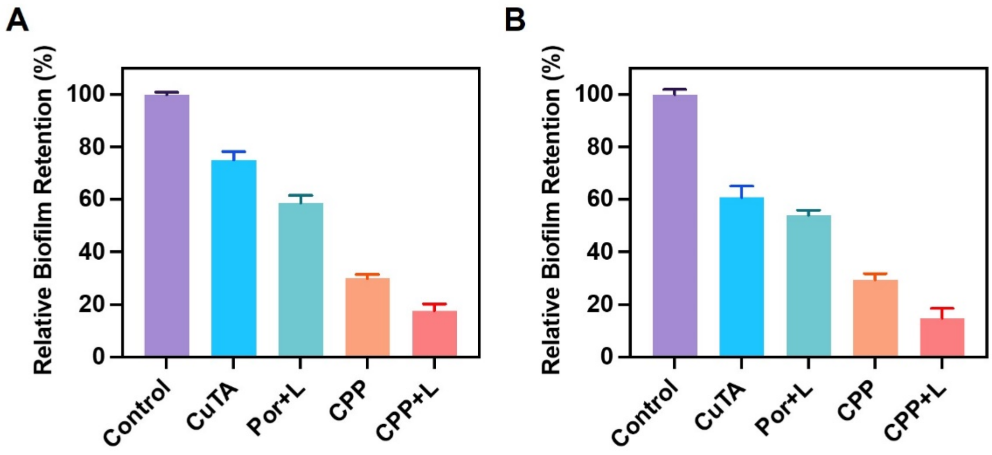

Figure S7. Quantitative analysis of the crystal violet-stained (A) established biofilms and (B) forming biofilms treated with different NPs.

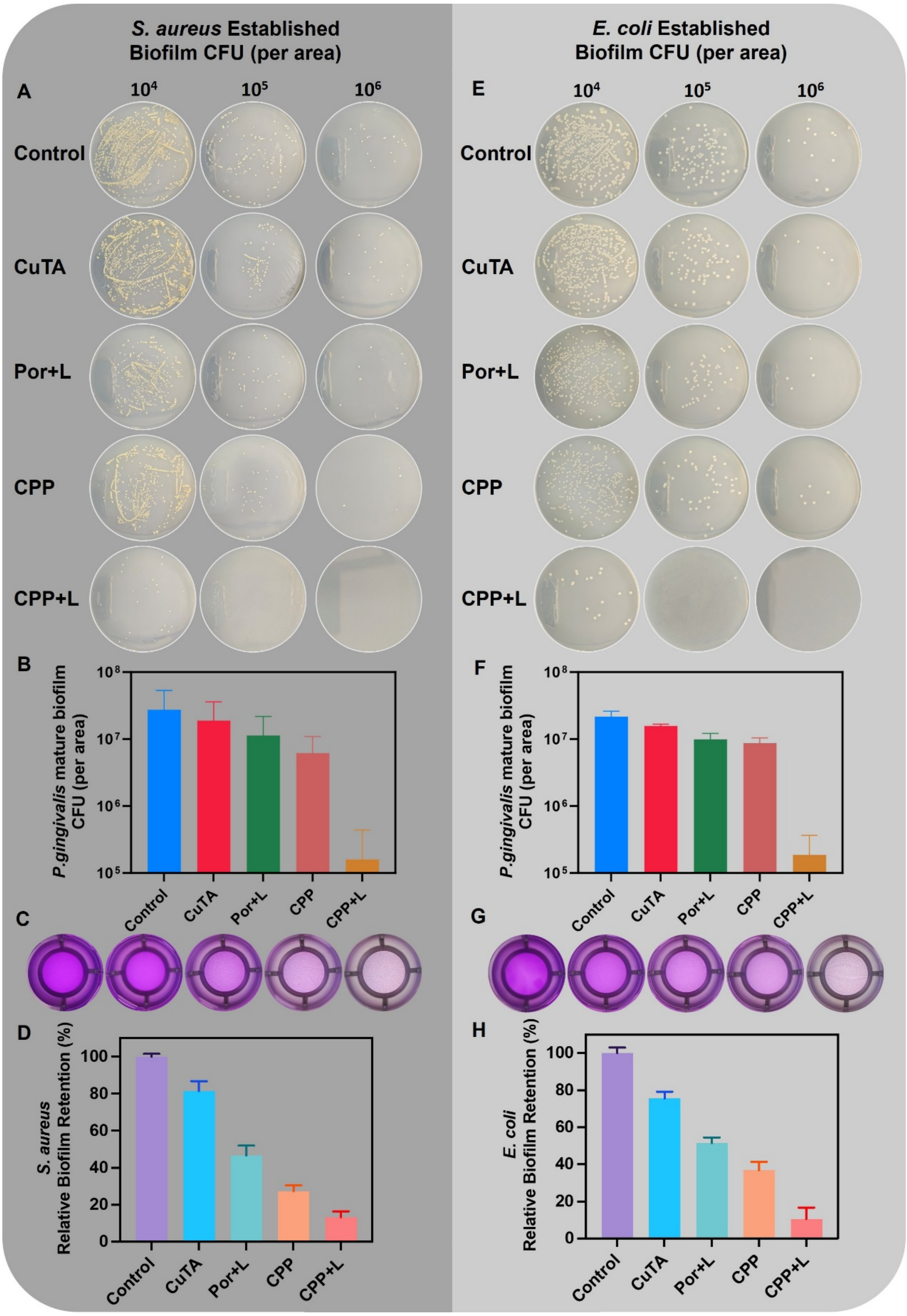

Figure S8. Antibacterial effects on established biofilms of *S. aureus* and *E. coli.* (A) Images of *S. aureus* colonies and (B) quantitative analysis. (C) Images and (D) corresponding quantitative analysis of the *S. aureus* biofilm after crystal violet staining.

(E) Images of *E. coli.* colonies and (F) quantitative analysis. (G) Images and (H) corresponding quantitative analysis of the *E. coli.* biofilm after crystal violet staining.

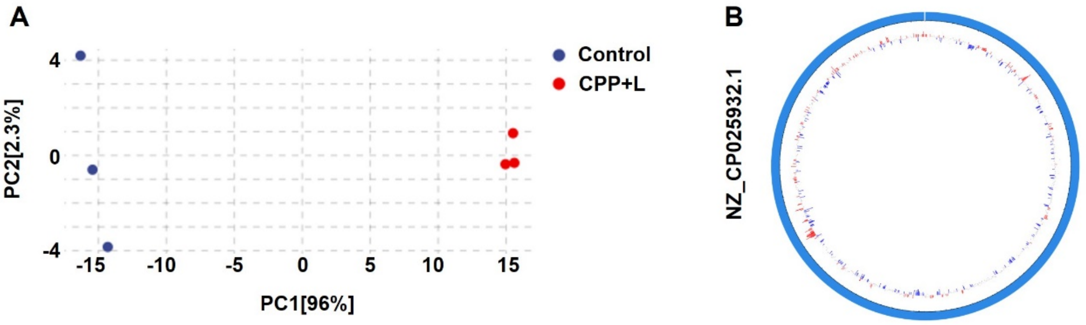

Figure S9. (A) Principal component analysis was performed based on DEGs in the *P. gingivalis* biofilm in Control and CPP+L groups. (B) Genomic cycle map based on DEGs in the *P. gingivalis* biofilm in Control and CPP+L groups.

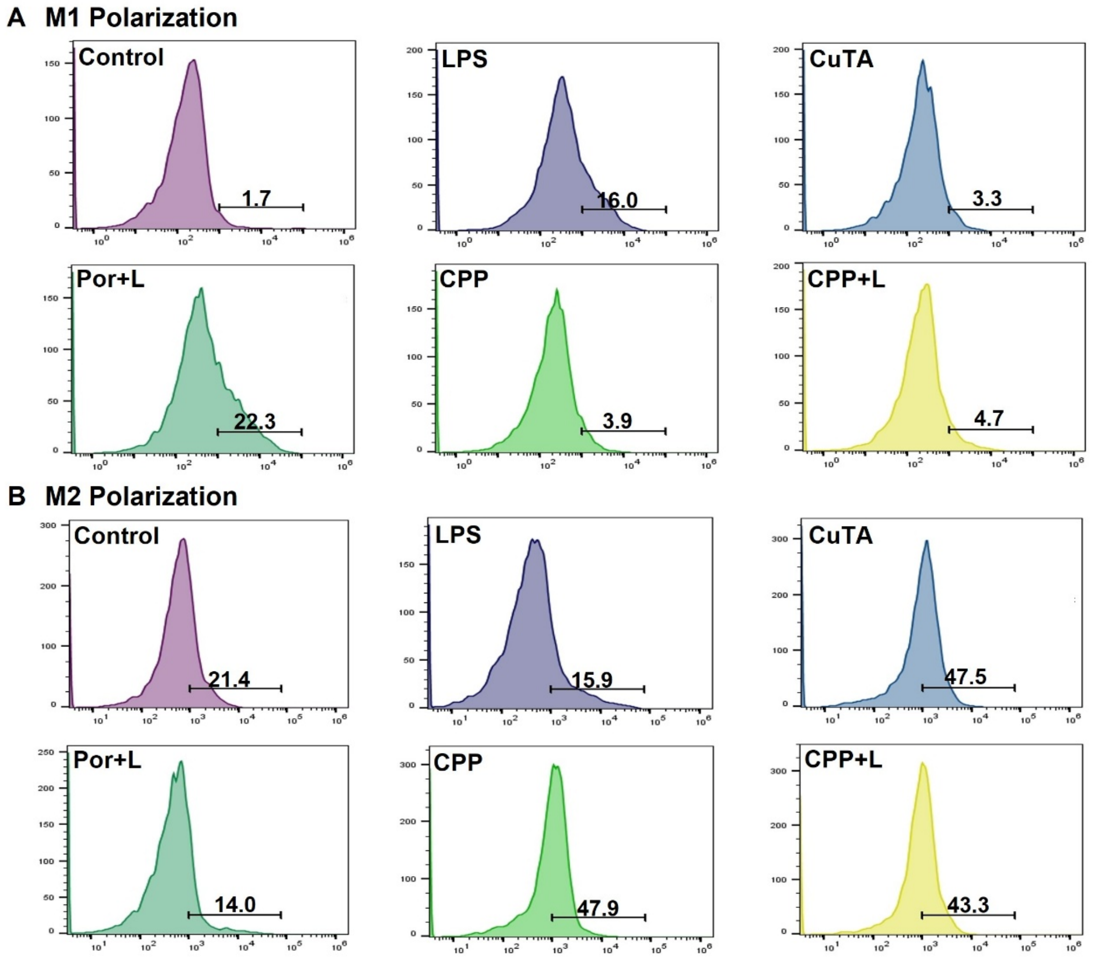

Figure S10. (A) Flow cytometry analysis of specific marker of M1 macrophage CD86

and (B) specific marker of M2 macrophage CD206.

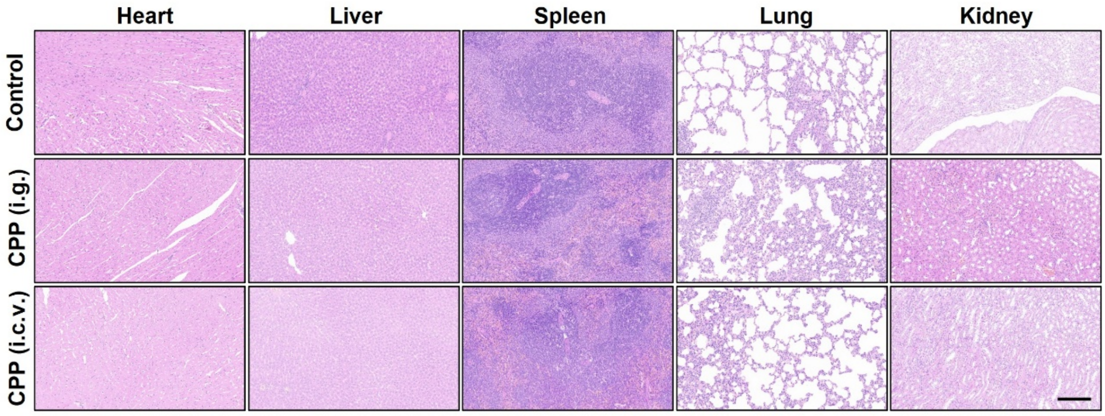

Figure S11. H&E staining of major organs of rats after different treatments. Scale bar: 200 μm.

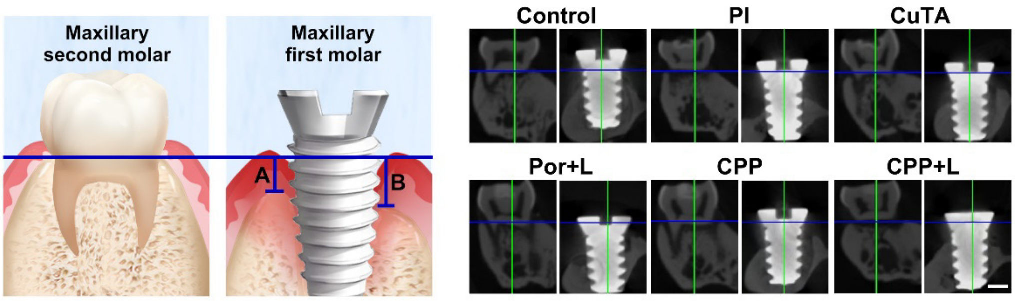

Figure S12. Scheme of the measurement of bone resorption height. Scale bar: 1 mm.

Table S1. Primer sequences used in this study.

Gene	Forward sequence (5' to 3')	Reverse sequence (5' to 3')
16s rRNA	TGTAGATGACTGATGGTGAAA	ACTGTTAGCAACTACCGATGT
Rgp A	CTGCGAGCGGTATTAGTGGT	CTACCAGCCCGTTTCCAACT
Rgp B	TCGGGACAAGTGTACGAACG	AACCAGTCTTGGGCTTCTCC
Kgp	AGCTGACAAAGGTGGAGACCAA AGG	TGTGGCATGAGTTTTTCGGAACCG T
Fim II	ACAACTATACTTATGACAATGG	AACCCCGCTCCCTGTATTCCGA
Fim IV	CTATTCAGGTGCTATTACCCAA	AACCCCGCTCCCTGTATTCCGA

Table S2. Primer sequences used in this study.

Gene	Forward sequence (5' to 3')	Reverse sequence (5' to 3')
β-Actin	CATCCGTAAAGACCTCTATGCCAAC	ATGGAGCCACCGATCCACA

IL-1β	TCCAGGATGAGGACATGAGCAC	GAACGTCACACACCAGCAGGTTA
IL-6	CCACTTCACAAGTCGGAGGCTTA	CCAGTTTGGTAGCATCCATCATTTC
TNF-α	ACTCCAGGCGGTGCCTATGT	GTGAGGGTCTGGGCCATAGAA
IL-10	CCAGTACAGCCGGGAAGACA	GAAGGCAGTCCGCAGCTCTA
TGF-β	CTTCAGCCTCCACAGAGAAGAACT	TGTGTCCAGGCTCCAAATATAG
Arg-1	TCATGGAAGTGAACCCAACTCTTG	TCAGTCCCTGGCTTATGGTTACC

Table S3. Routine blood test of rats after 7 days of treatments.

	**Normal range**	**Control**	**CPP (i.g.)**	**CPP (i.c.v.)**
**WBC**	1.90-16.80 (10^9/L)	4.49±0.35	4.66±0.40	4.61±0.56
**Neu#**	0.35-6.30 (10^9/L)	0.52±0.04	0.47±0.33	0.55±0.07
**Lym#**	0.91-12.20 (10^9/L)	3.50±0.25	3.94±1.92	3.80±0.49
**Mon#**	0.08-2.30 (10^9/L)	0.33±0.03	0.17±0.10	0.20±0.00
**Eos#**	0.00-0.60 (10^9/L)	0.11±0.06	0.08±0.04	0.06±0.01
**Bas#**	0.00-0.10 (10^9/L)	0.04±0.01	0.01±0.01	0.00±0.00
**Neu%**	7.30-50.00 (%)	11.55±0.05	8.65±2.65	11.80±0.20
**Lym%**	40.00-88.90 (%)	77.90±0.60	86.15±1.15	82.50±0.70
**Mon%**	2.00-18.00 (%)	7.40±1.10	3.35±0.55	4.20±0.50
**Eos%**	0.50-6.00 (%)	2.35±0.95	1.75±0.05	1.45±0.45
**Bas%**	0.00-1.00 (%)	0.80±0.08	0.10±0.00	0.05±0.05
**RBC**	5.00-9.80 (10^{12}/L)	5.89±0.81	7.91±0.66	7.47±0.15
**HGB**	120.00-170.00 (g/L)	125.50±8.50	155.00±3.00	146.50±2.50
**HCT**	32.00-53.00 (%)	39.65±5.25	50.70±2.30	48.00±0.30
**MCV**	50.00-67.00 (fL)	67.35±0.35	64.25±2.45	64.30±0.80
**MCH**	16.00-23.00 (pg)	21.55±1.55	19.70±1.30	19.60±0.00
**MCHC**	300.00-370.00 (g/L)	320.00±21.00	306.00±8.00	305.00±4.00
**RDW-CV**	11.00-16.00 (%)	15.45±0.15	12.90±0.20	13.90±0.10
**RDW-SD**	30.00-50.00 (fL)	39.05±0.45	31.65±1.95	34.00±0.65
**PLT**	250.00-1500.00 (10^9/L)	840.00±98.00	871.50±10.50	962.50±30.50
**MPV**	4.80-7.50 (fL)	10.85±0.55	9.40±1.00	9.20±0.20
**PDW**	12.00-17.50	15.90±0.10	15.75±0.15	15.65±0.15
**PCT**	0.20-0.78 (%)	0.71±0.06	0.72±0.09	0.79±0.01

Table S4. Liver and kidney functions test of rats after 7 days of treatments.

	**Normal range**	**Control**	**CPP (i.g.)**	**CPP (i.c.v.)**
**Liver Function**				
**ALT**	21.53-61.75 (U/L)	30.62±3.16	35.06±4.77	31.61±1.12
**AST**	41.47-195.65 (U/L)	104.40±18.00	176.67±0.95	159.39±7.74
**ALB**	21.16-34.77 (g/L)	33.76±0.26	38.97±1.98	33.72±0.76
**ALP**	12.04-610.97 (U/L)	198.44±67.21	229.96±66.64	103.54±2.27
**γ-GT**	0.58-6.81 (U/L)	4.53±0.18	3.07±1.41	4.55±0.03
**DBIL**	2.24-16.892 (μM)	9.27±1.49	10.08±2.17	6.68±0.71
**TBIL**	2.57-36.85 (μM)	21.11±7.45	24.47±5.34	19.62±0.25

**TBA**	9.03-14.54 (μM)	12.77±0.23	12.14±1.39	12.20±0.65
**Renal Function**				
**BUN**	9.75-22.71 (mg/dL)	16.79±3.59	18.07±1.73	14.14±0.04
**CREA**	10.90-118.07 (mM)	26.54±6.42	27.25±7.70	20.96±2.09
**UA**	58.38-122.65 (μM)	68.77±3.85	76.97±6.45	71.52±4.21
